# Supplementary figures and images for: Structures of three MORN repeat proteins and a re-evaluation of the proposed lipid-binding properties of MORN repeats
Source: PLoS One. 2020 Dec 9;15(12):e0242677. doi: 10.1371/journal.pone.0242677 (PMC7725318; doi:10.1371/journal.pone.0242677)

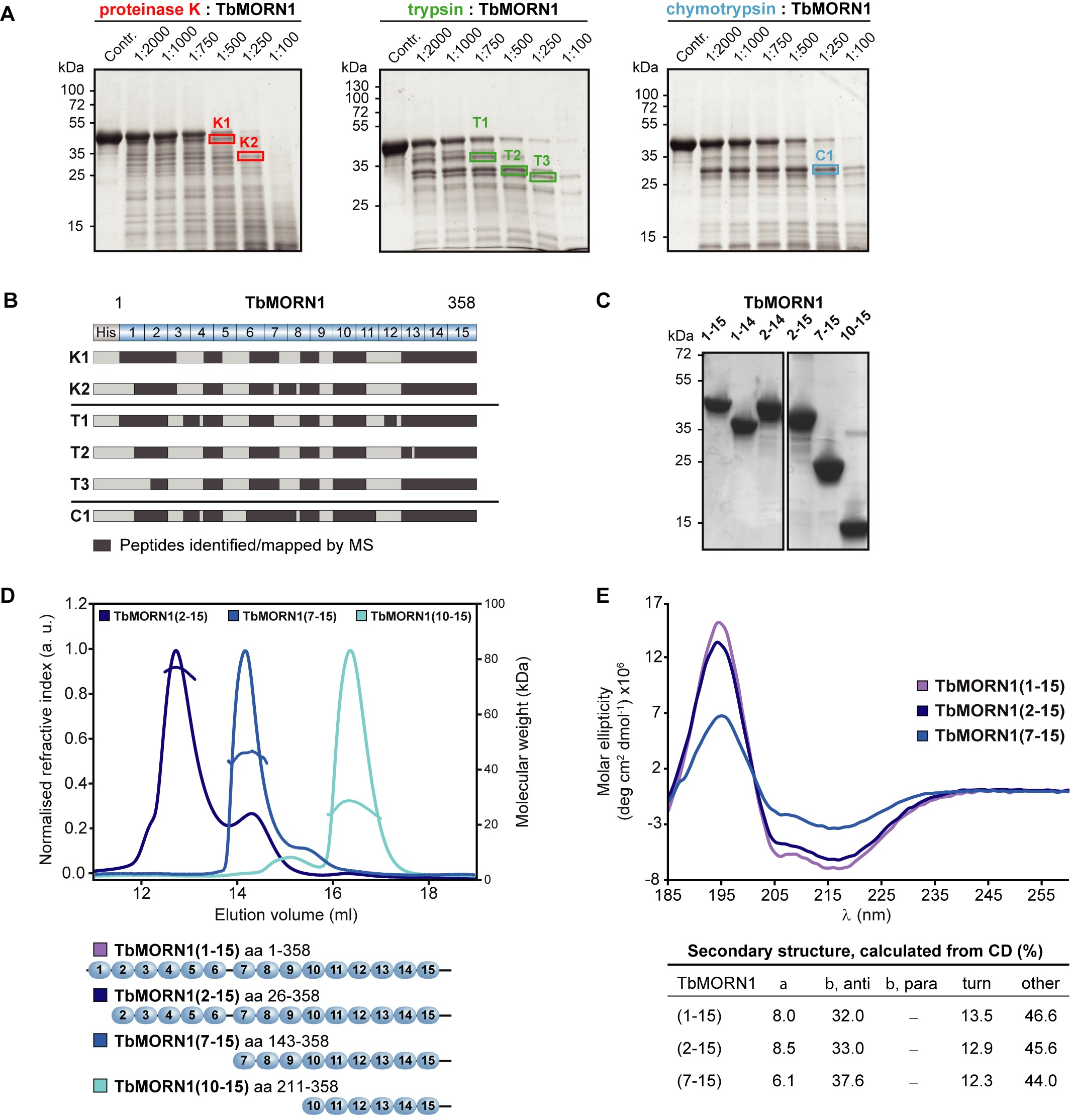

Supplement: S1 Fig — (A) Full-length TbMORN1 with an N-terminal HisTag was subjected to limited proteolysis using proteinase K (red), trypsin (green), and chymotrypsin (cyan) at protease:protein ratios (w/w) as indicated on each panel. Samples were resolved by SDS-PAGE and selected bands (labelled boxes) corresponding to proteolytic products were excised and analysed by mass spectrometry. Control (Contr.) corresponds to protein without protease treatment. (B) Mass spectrometry analysis of the excised proteolytic products indicated in panel A. Peptides identified and mapped by mass spectrometry are shown as dark grey boxes; a schematic of the full-length construct is shown above, with individual MORN repeats labelled. Note that the proteolytic products show progressive degradation from their N-termini, while the C-terminal part is stable. (C) Coomassie-stained SDS-PAGE gel showing purified recombinant TbMORN1 truncations. (D) SEC-MALS traces of TbMORN1(2–15), (7–15), and (10–15). Chromatographic separation was done using a Superdex 200 Increase 10/300 GL column, void volume 7.2 ml. The three proteins all eluted as dimers. Schematics are shown underneath. (E) Far-UV CD profiles of TbMORN1, TbMORN1(2–15) and (10–15). A positive peak at 195 nm and a negative one at 218 nm demonstrated that the constructs are all β-proteins. The secondary structure content predictions for each construct were calculated in BeStSel and are shown below the CD graph. (TIF) [file pone.0242677.s001.tif]

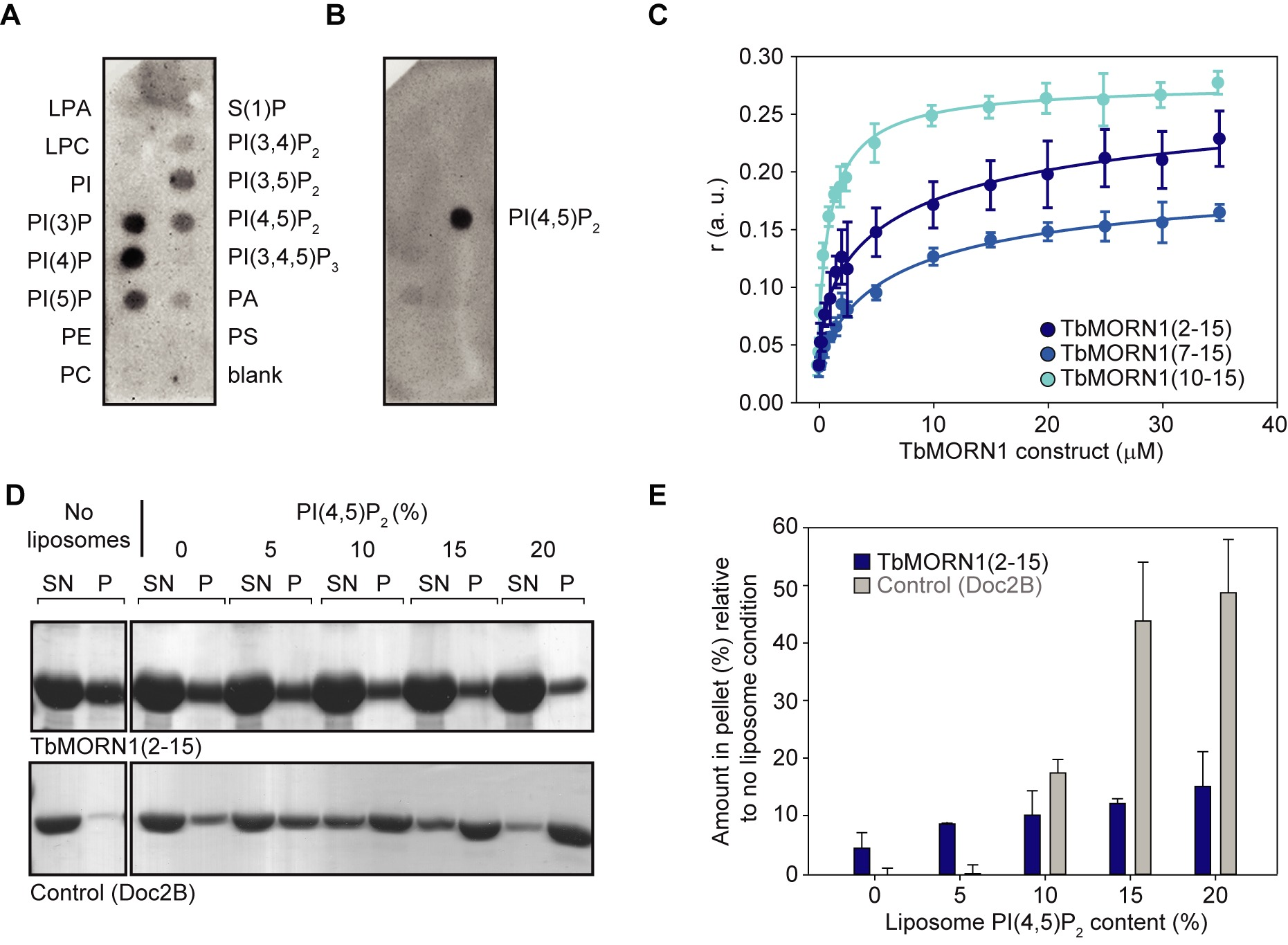

Supplement: S2 Fig — (A) Purified recombinant TbMORN1 binds to multiple phospholipid species in protein-lipid overlay assays. PIP strips were incubated with purified recombinant TbMORN1(1–15) protein, and bound proteins were detected by immunoblotting with anti-strep antibodies. Abbreviations: PI(n)P, phosphatidylinositol (n) phosphate; PA, phosphatidic acid; LPA, lysophosphatidic acid; LPC, lysophosphatidylcholine; PI, phosphatidylinositol; PE, phosphatidylethanolamine; PC, phosphatidylcholine; S(1)P, sphingosine-1-phosphate; PS, phosphatidylserine. Data were obtained from 3 independent experiments using 2 biological replicates; an exemplary blot is shown. (B) PIP strip overlaid with the PH domain of PLCδ, a positive control for PI(4,5)P2 binding. Data were obtained from 3 independent experiments using 3 biological replicates; an exemplary blot is shown. The PIP strips presented here were exposed to the light source for the same time. (C) Fluorescence anisotropy measurements of 0.1 μM BODIPY TMR-PI(4,5)P2-C16 in the presence of TbMORN1(2–15), (7–15) and (10–15). All three truncations of TbMORN1 interacted with the 16-carbon PI(4,5)P2 with binding affinities in the low micromolar range. Data obtained from 3 independent experiments using 3 biological replicates, with 10 technical replicates for each experiment. Traces show mean values, bars are s.e.m. (D) Liposome co-sedimentation assay. POPC liposomes containing 0, 5, 10, 15 and 20% of porcine brain PI(4,5)P2 were incubated with 10 μM TbMORN1(2–15). TbMORN1(2–15) was found in both pellet (P) and supernatant (SN) fractions but did not increase proportionally to PI(4,5)P2 concentration. The positive control, Doc2B, bound PI(4,5)P2 in a concentration-dependent manner, with a shift from SN to P fractions proportional to the increase in % of PI(4,5)P2 present in the liposomes. Data were obtained from 2 independent experiments using 2 biological replicates; an exemplary blot is shown. (E) Quantification of the liposome pelleting assays. The a [file pone.0242677.s002.tif]

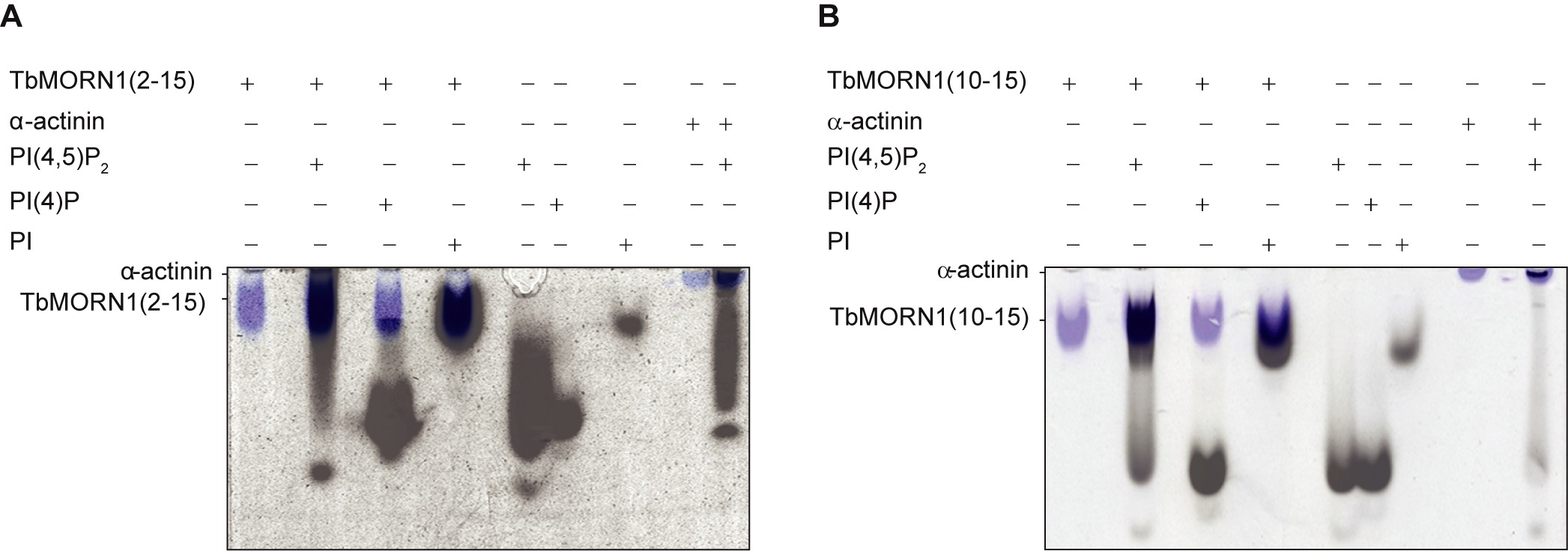

Supplement: S3 Fig — (A) Native gel electrophoresis of TbMORN1(2–15) and (B) TbMORN1(10–15) in the presence and absence of PI(4,5)P2, PI(4)P and PI, all labelled with BODIPY TMR fluorescent dye. α-actinin served as a positive control of PI(4,5)P2 binding. Data obtained from two independent experiments, each using a different biological replicate. (TIF) [file pone.0242677.s003.tif]

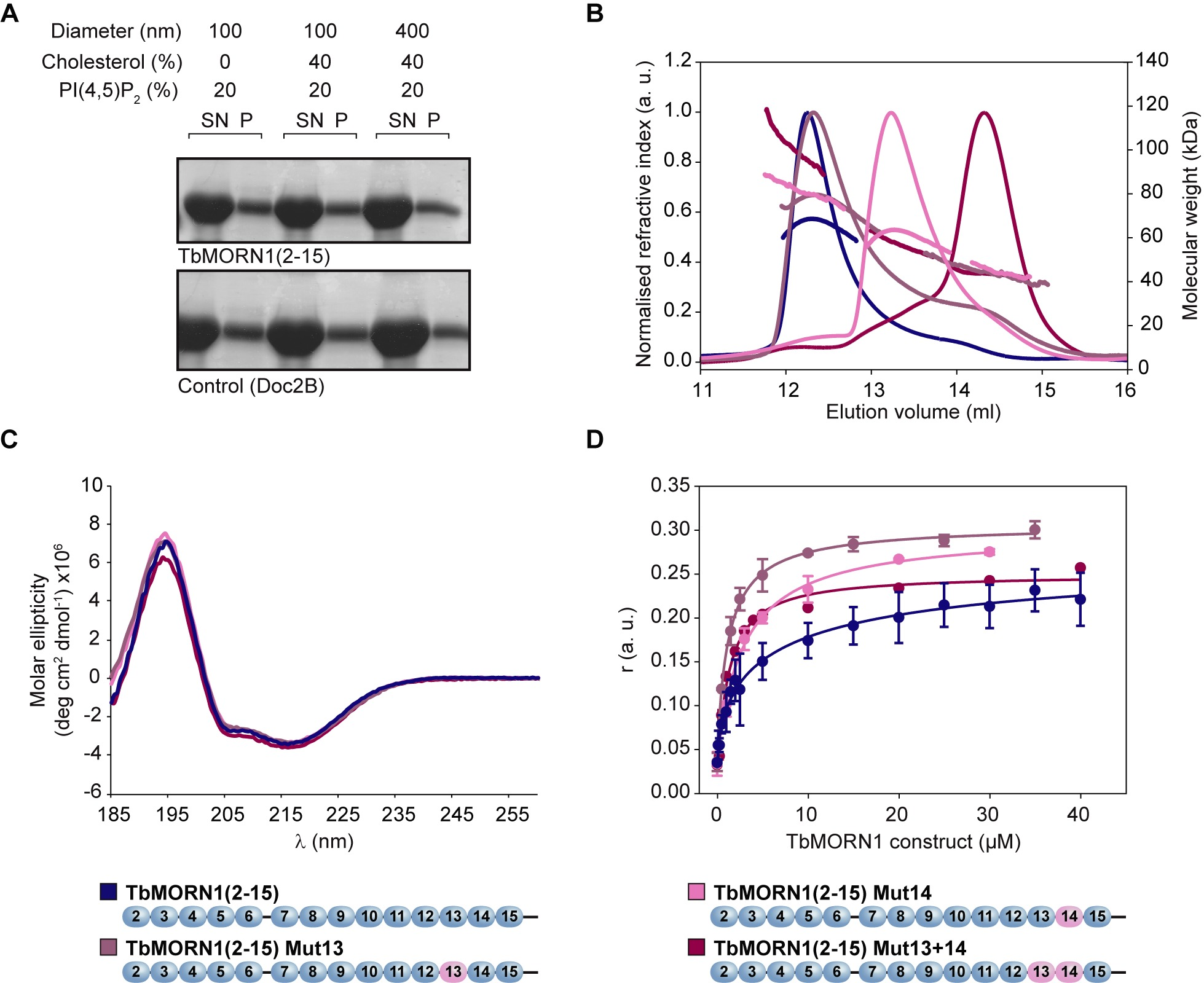

Supplement: S4 Fig — (A) Liposome co-sedimentation assay performed on TbMORN1(2–15) in the presence of POPC liposomes containing 20% of porcine brain PI(4,5)P2 and 0 or 40% cholesterol. The excess cholesterol was expected to promote local high concentrations of PI(4,5)P2 on the surface of the liposomes. To assay for the effect of curvature, two batches of liposomes containing 20% PI(4,5)P2 and 40% of cholesterol were tested, with the diameter of the liposomes being either 100 or 400 nm. No significant co-sedimentation of TbMORN1(2–15) and PI(4,5)P2-containing liposomes was observed. The positive control, 10 μM Doc2B was predominantly found in the pellet (P) fractions. (B) SEC-MALS profiles of TbMORN1(2–15) and its mutagenised variants. Residues comprising the putative PI(4,5)P2-binding sites in MORN repeats 13 and 14 were mutated to alanines. Mutagenesis of repeat 13 (Mut13) did not result in any change to the dimeric status of the protein. However, mutagenesis of repeat 14 (Mut14) resulted in a mixture of monomers and dimers being eluted, while mutagenesis of both repeats (Mut13+14) resulted in monomeric protein. Chromatographic separation was done using a Superdex 200 Increase 10/300 GL column, void volume 7.2 ml. (C) Far-UV CD profiles of TbMORN1(2–15) and its putative PI(4,5)P2-binding mutants. The constructs remained β-proteins despite the site-directed mutagenesis. (D) Fluorescence anisotropy measurements of TbMORN1(2–15) and its putative PI(4,5)P2-binding mutants, measured in the presence of 0.1 μM BODIPY TMR-PI(4,5)P2. All constructs showed good interaction with the fluorophore-conjugated PI(4,5)P2. (TIF) [file pone.0242677.s004.tif]

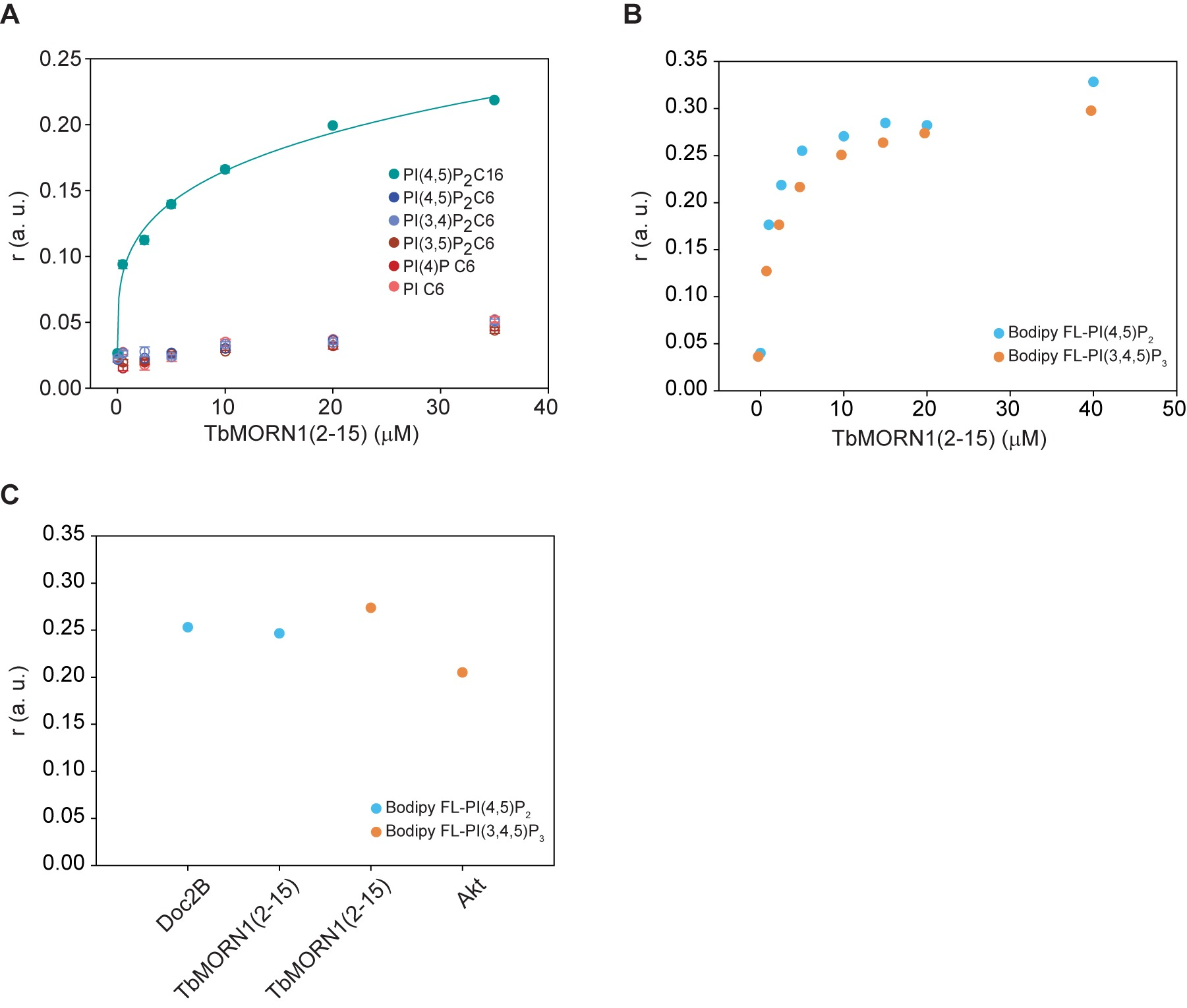

Supplement: S5 Fig — (A) Fluorescence anisotropy measurements of TbMORN1(2–15) in the presence of BODIPY TMR-labelled PI(4,5)P2 C16, PI(4,5)P2 C6, PI(3,4)P2 C6, PI(3,5)P2 C6, PI(4)P C6 and PI C6. Binding was only observed with the 16-carbon PI(4,5)P2. (B) Fluorescence anisotropy measurements of TbMORN1(2–15) measured in the presence of BODIPY Fluorescein-labelled PI(4,5)P2 C16 and PI(3,4,5)P3 C16. Both 16-carbon lipids bound equally well. (C) Comparison of TbMORN1(2–15) binding to 16-carbon PI(4,5)P2 and PI(3,4,5)P3 with two positive controls, respectively Doc2B and Akt. (TIF) [file pone.0242677.s005.tif]

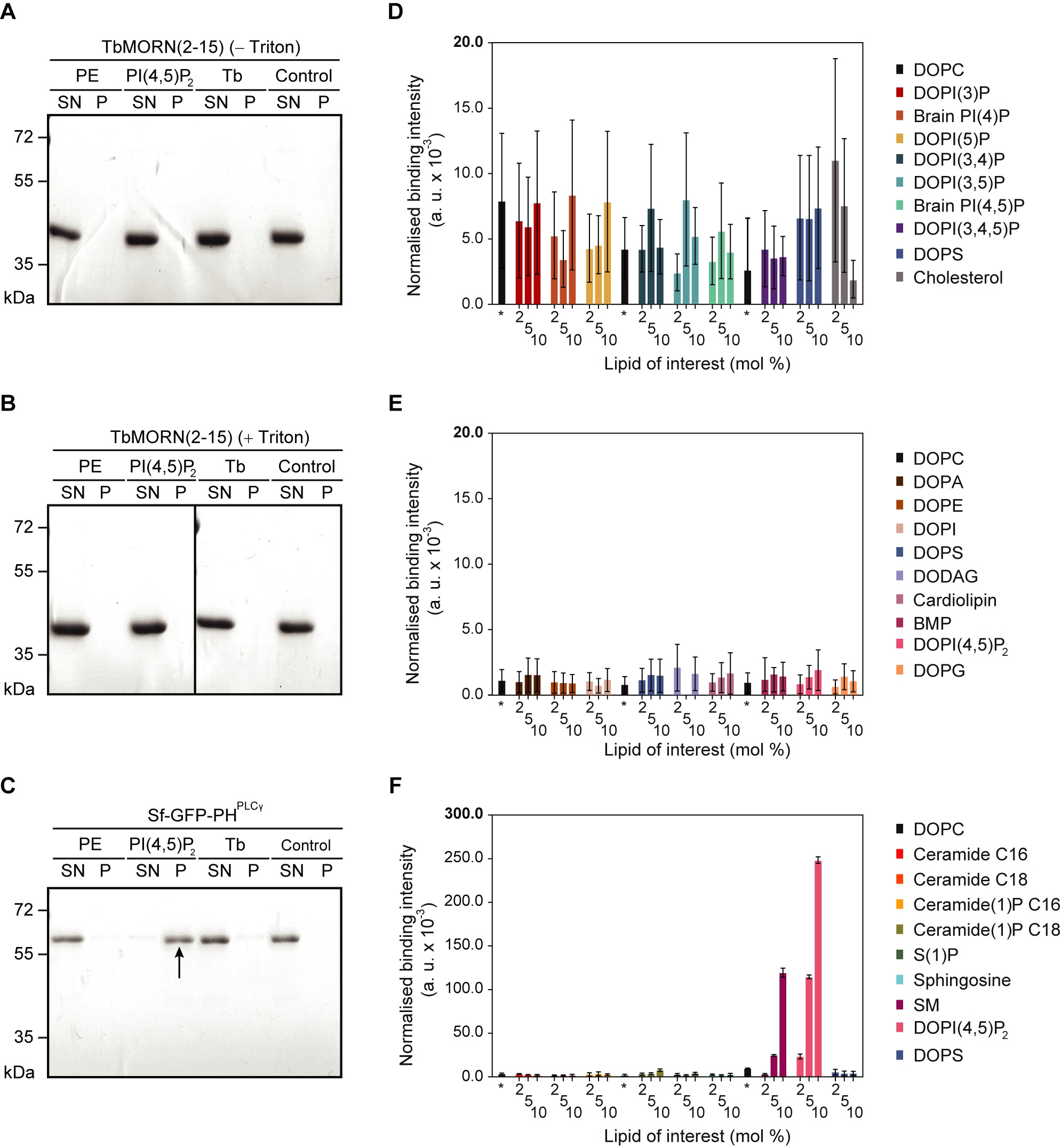

Supplement: S6 Fig — (A-C) Liposome pelleting assays using sucrose-loaded vesicles (SLVs). TbMORN1(2–15) was purified in the absence (A) or presence (B) of Triton X-100 in the lysis buffer. The purified proteins were incubated with SLVs, which were then pelleted by centrifugation. Supernatant (SN) and pellet (P) fractions were analysed by SDS-PAGE using Coomassie staining. The SLVs were made from commercial lipids with an excess of either PE or PI(4,5)P2, and also reconstituted from purified whole-cell trypanosome lipids (Tb). A no-SLV condition was included as an additional negative control. (C) The PH domains of PLCγ was used as a positive control for PI(4,5)P2 binding. As expected, the PLCγ PH domain co-sedimented with PI(4,5)P2-containing SLVs and was entirely present in the P fraction in this condition (arrow). The recombinant TbMORN1 proteins remained in the SN fraction in all conditions. (D-F) Liposome microarray analysis. Microchips carrying giant unilamellar vesicles (GUVs) with lipids of interest at three different concentrations (2, 5 and 10 mol %) were incubated with purified recombinant EGFP-TbMORN1(2–15). No significant binding was observed to either phosphoinositide lipids or PE. (D) n (independent replicates) = 7, bars show standard deviation. (E) n (independent replicates) = 3, bars show standard deviation. (F) Microchip incubated with PLC-δ1 PH domain as a positive control. A specific and concentration-dependent binding between the PLC-δ1 PH domain and DOPI(4,5)P2 and SM was observed. DOPC, a carrier lipid, was used as an internal negative control of binding, as well as a marker for tracking positions of liposomes on a given microarray. n (independent replicates) = 1, bars show standard deviation. Note the different scales on the y-axes of the three charts. (TIF) [file pone.0242677.s006.tif]

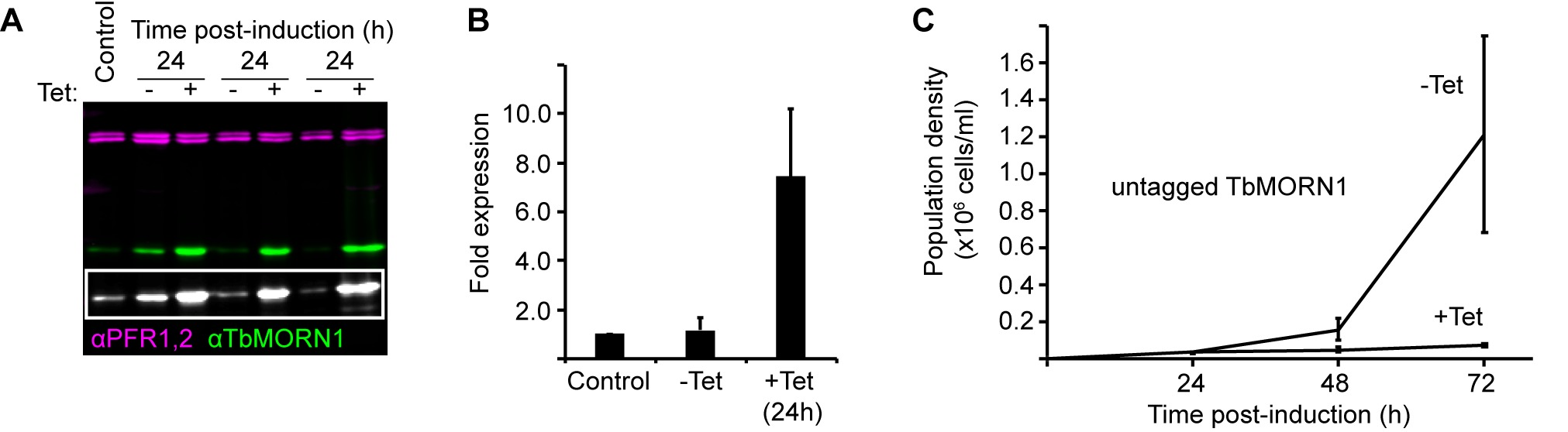

Supplement: S7 Fig — (A) Inducible overexpression of untagged TbMORN1. Immunoblot of whole-cell lysates from three separate clones overexpressing untagged TbMORN1 from an ectopic locus. TbMORN1 was detected using anti-TbMORN1 antibodies; PFR1,2 were used as a loading control and detected using anti-PFR1,2 antibodies. Inset shows a greyscale image of the TbMORN1 channel with enhanced levels so that the endogenous protein is visible. Three separate clones were assayed, each in three independent experiments; an exemplary blot is shown. One of the three clones appeared to have slightly leaky expression, with TbMORN1 levels in the -Tet condition being higher than controls. (B) Quantification of overexpression. TbMORN1 levels in control, uninduced (-Tet) and induced (+Tet) were normalised relative to the loading control and expressed relative to the control cells. Approximately 7-fold overexpression was achieved relative to control cells. Data were obtained from blots using 3 separate clones, each induced in 3 independent experiments. Bars show mean + SD. (C) Overexpression of untagged TbMORN1 is deleterious. Uninduced (-Tet) and TbMORN1 overexpressing (+Tet) cells were assayed at 24 h intervals in a 3-day time course. Data were obtained from blots using 3 separate clones, each induced in 3 independent experiments. Mean +/- SD. (TIF) [file pone.0242677.s007.tif]

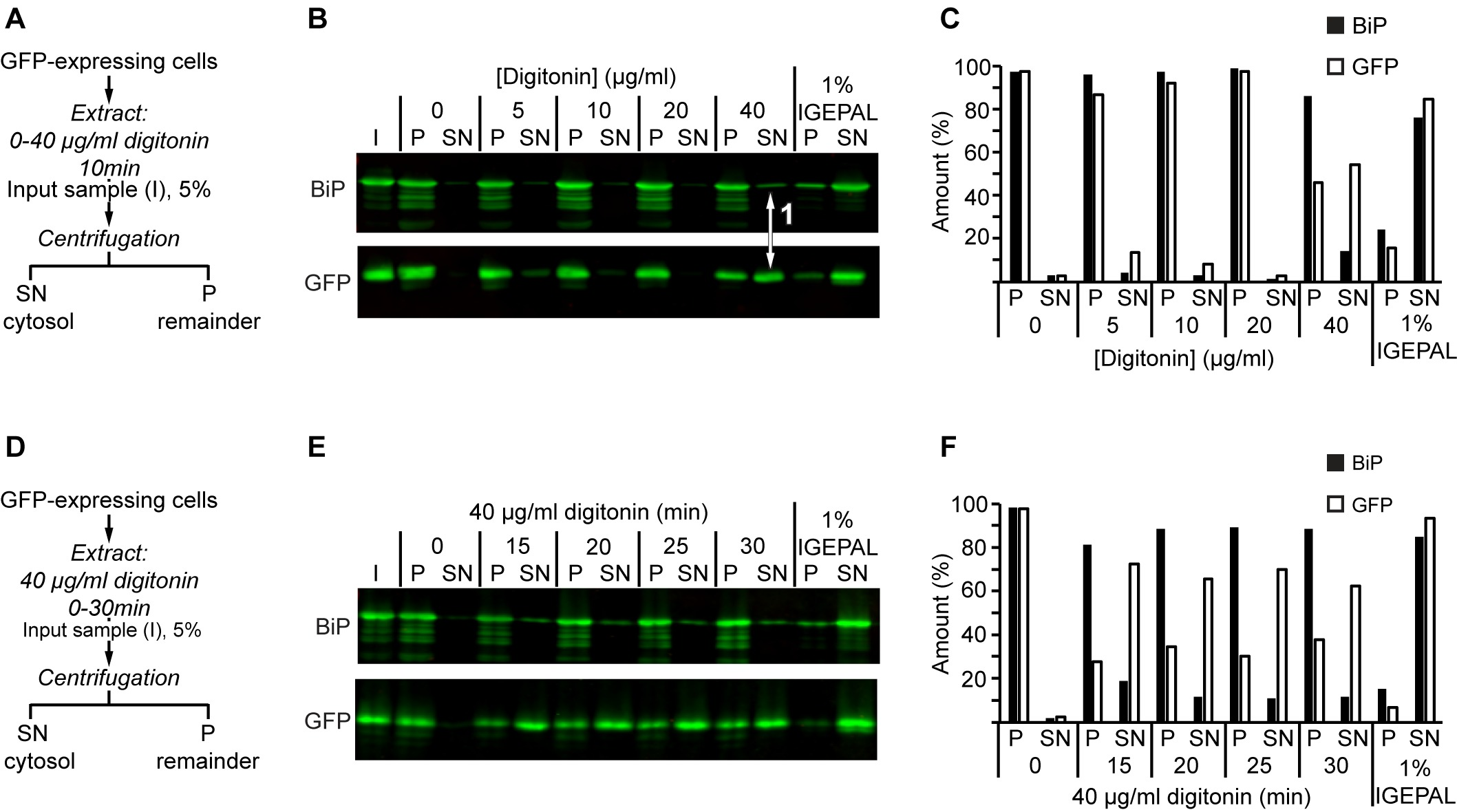

Supplement: S8 Fig — (A) Schematic of the one-step fractionation scheme. Cells expressing cytosolic GFP (Batram et al., 2014) were incubated for 10 min with increasing concentrations (0–40 μg/ml) of digitonin, using 1% IGEPAL as a positive control for full extraction. At the end of the incubation, the soluble and insoluble fractions were separated by centrifugation. (B) Equal fractions (5%) of the supernatant (SN) and pellet (P) were probed with antibodies specific for GFP and the endoplasmic reticulum chaperone BiP. At 40 μg/ml digitonin, good solubilisation of GFP was achieved with only negligible solubilisation of BiP (arrow 1). Both proteins were efficiently solubilised using 1% IGEPAL as a positive control. Multiple independent experiments were carried out; an exemplary blot is shown. Note that the membrane was cut into strips prior to blotting, but the samples shown are from the same experiment. (C) Quantification of the immunoblot shown in B. (D, E, F) As per panels A-C, but with constant 40 μg/ml digitonin concentration and varying incubation time (0–30 min). Increasing the incubation time over a range of 15–30 min did not appear to notably increase the amount of GFP extraction. As a result, a 25 min incubation time was used in the subsequent experiments. (TIF) [file pone.0242677.s008.tif]

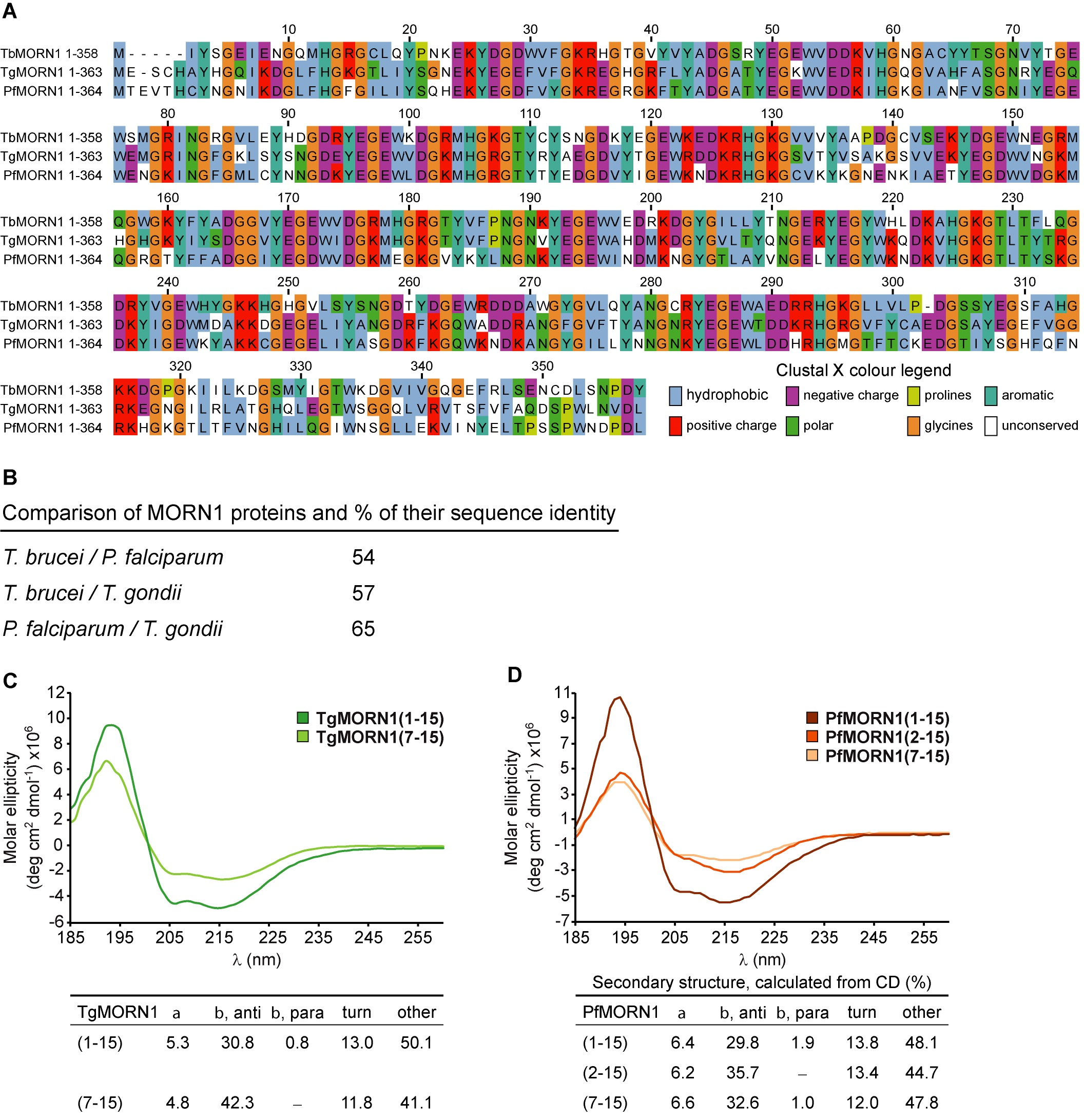

Supplement: S9 Fig — (A) Amino acid sequence alignment of MORN1 proteins from Trypanosoma brucei, Toxoplasma gondii and Plasmodium falciparum. The number of amino acids in each protein is indicated, amino acid numbers in the alignment are those for TbMORN1. The alignment is coloured according to the amino acid properties. (B) Pairwise comparison of percentage sequence identity between the three proteins. (C) Far-UV CD measurements obtained for TgMORN1(1–15) and TgMORN1(7–15). The secondary structure content predictions for each of the measured proteins were calculated in BeStSel and are shown below the CD graph. (D) As (C), but PfMORN1(1–15), (2–15) and (7–15). Like TbMORN1, TgMORN1 and PfMORN1 are also all-β proteins. (TIF) [file pone.0242677.s009.tif]

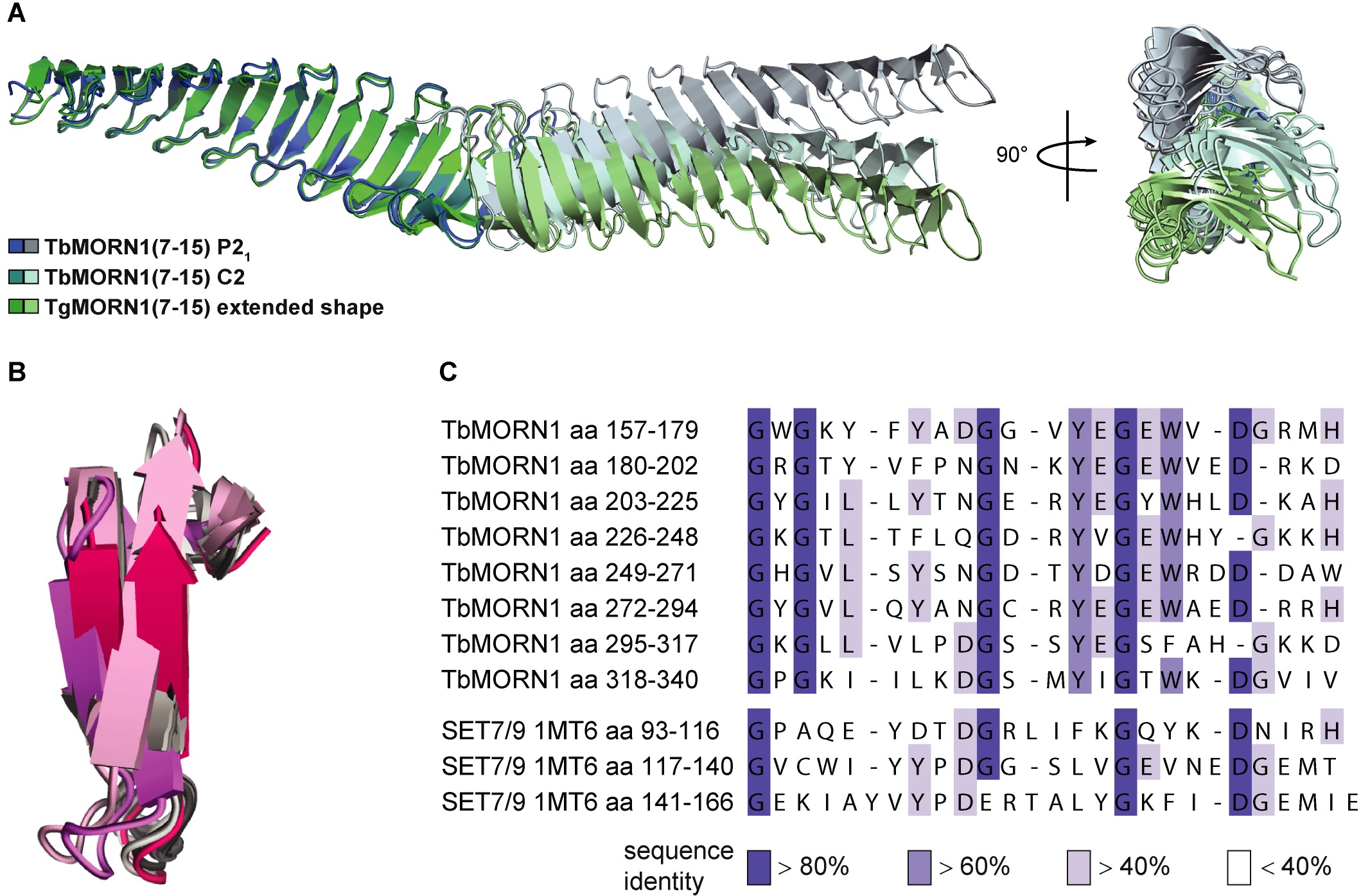

Supplement: S10 Fig — (A) TbMORN1(7–15) P21, TbMORN1(7–15) C2, and TgMORN1(7–15) extended dimers superimposed on each other and displayed in two orientations. In contrast to the other two proteins, the P21 crystal structure of TbMORN1(7–15) displays a bend of approximately 30° among the sub-units. (B) TbMORN1(7–15) MORN repeats superimposed on three MORN repeats from SETD7 (SET7/9). Alignment of the three MORN repeats from SETD7 with MORN repeat 7 from the TbMORN1(7–15) crystal structure over 22–23 aligned C-atoms yielded rmsd values of 2.3, 1.5 and 1.9 Å respectively. (C) Sequence alignment of MORN repeats from the TbMORN1(7–15) crystal structure with three MORN repeats from SETD7. The first Gly residue is conserved in all MORN repeats of TbMORN1(7–15) and SETD7 structures. (TIF) [file pone.0242677.s010.tif]

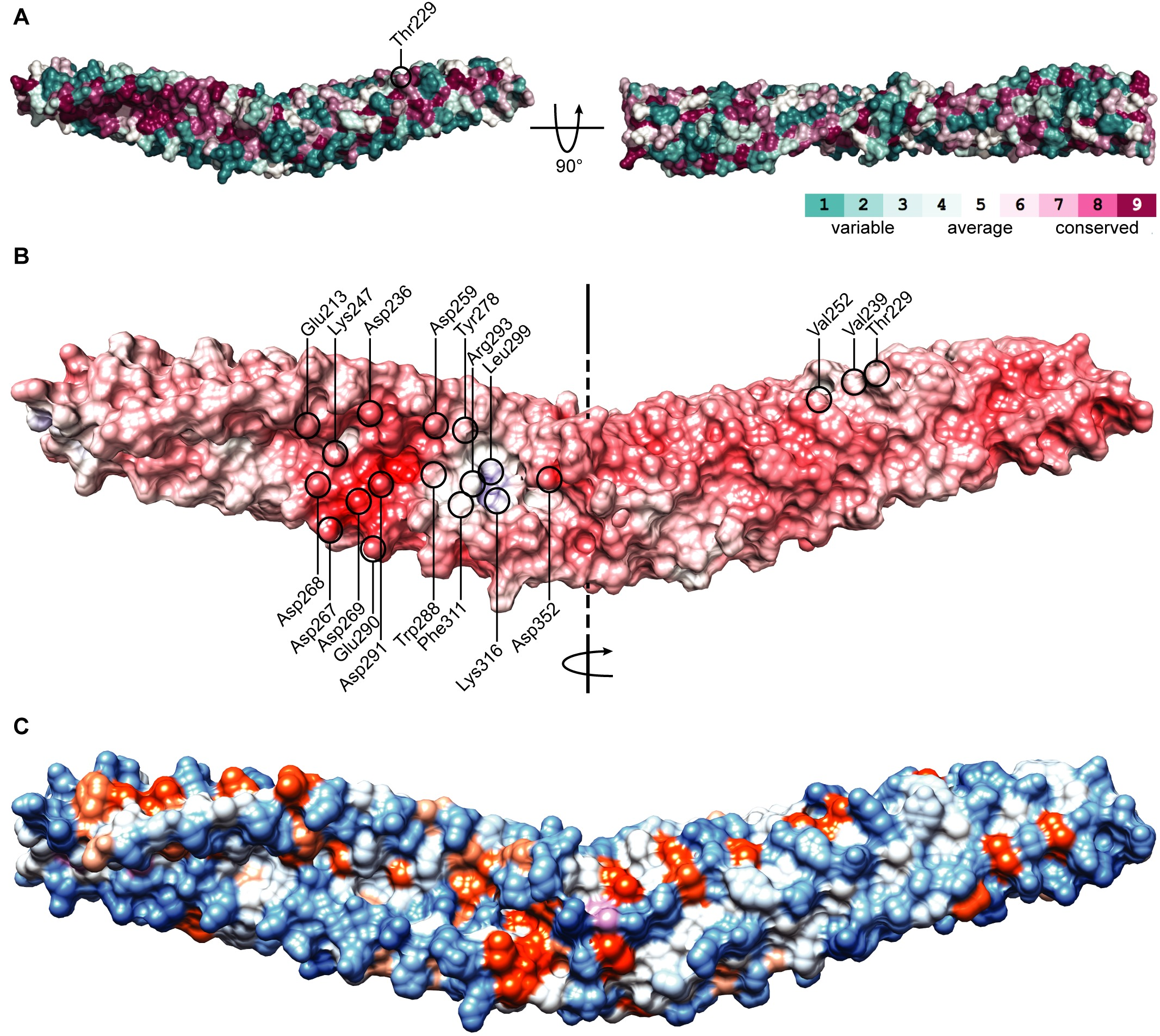

Supplement: S11 Fig — (A) Conservation map of the TbMORN1(7–15) P21 crystal structure reveals a highly conserved stretch of residues along the groove. The structure is shown in two orientations, with residues colour-coded according to the level of conservation. (B) An electrostatic map of TbMORN1(7–15) P21. Calculations were performed using APBS suite, displayed by Chimera. Colour scale: red = -13 kT; blue = +13 kT. Individual residues contributing to its surface electrostatics are labelled, namely those of the two negatively-charged loops building up a negative patch inside the groove, and the residues contributing to a small positively-charged region close to the dimer interface. (C) Hydrophobic map of TbMORN1(7–15) P21. Colour scale: blue = hydrophilic; orange = hydrophobic, pink = methionine residues. (TIF) [file pone.0242677.s011.tif]

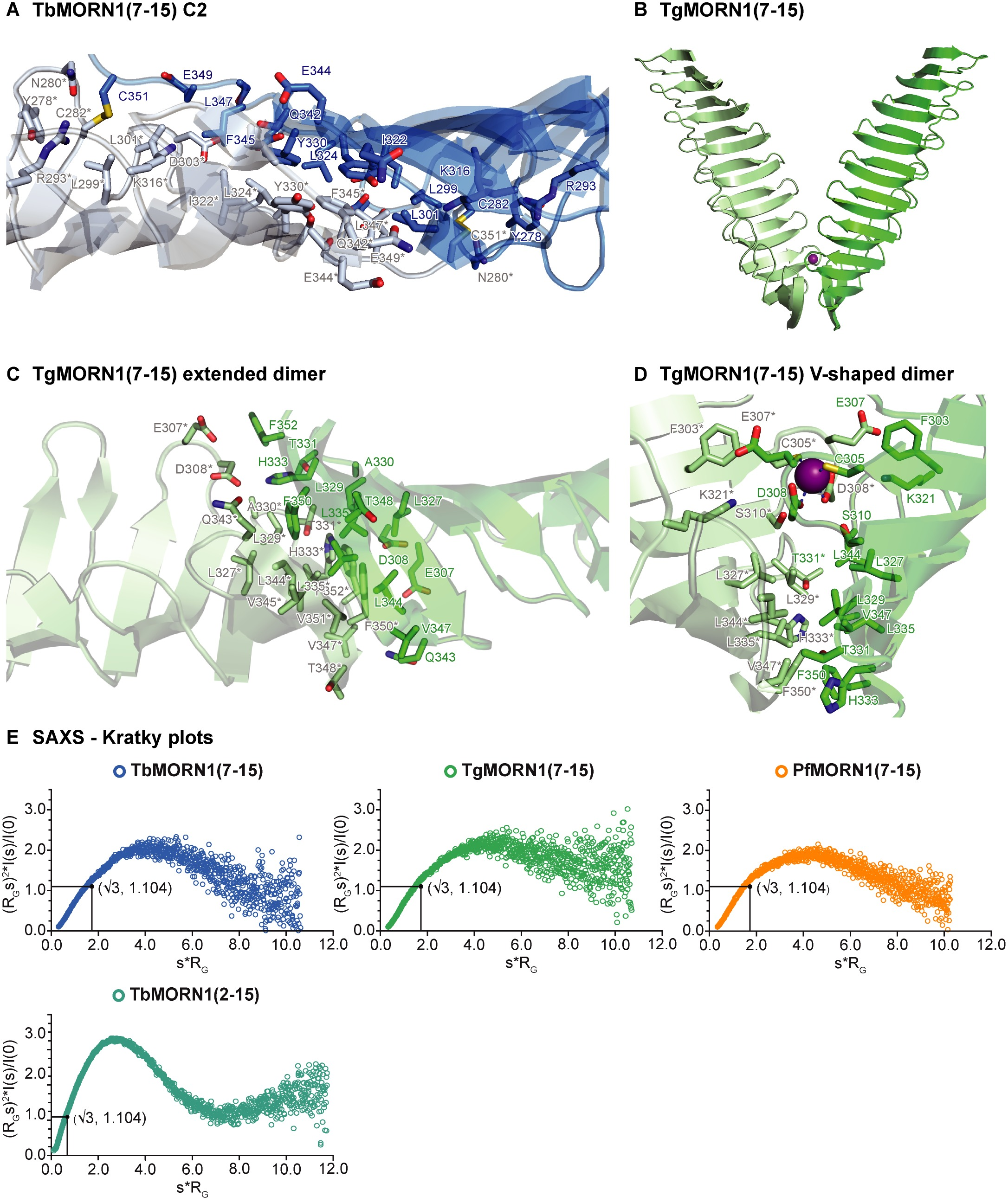

Supplement: S12 Fig — (A) Dimer interface of TbMORN1(7–15) C2 crystal form. In comparison to the P21 form, the dimer interface of C2 structure is broader, and is additionally stabilised by two disulphide bridges formed between Cys351 at the C-terminus of repeat 15 and Cys282 from the β-hairpin loop of repeat 12. (B) Crystal structure of the TgMORN1(7–15) V-shaped dimer, incorporating Zn2+ in its dimerisation interface. (C) Dimer interface of the TgMORN1(7–15) extended dimer, which utilises residues from MORN repeats 13–15. In contrast to TbMORN1(7–15), where the dimerisation interface is centred around aromatic stacking, a hydrophobic core plays a crucial role in the dimer interface of extended TgMORN1(7–15). Leu327, Leu329, Leu335, Leu344, Val345, Val347, Phe350 and Phe352 are part of this hydrophobic core. The dimer is stabilised by a single salt bridge formed between the Asp308 of one subunit and the His333 of the other subunit. This salt bridge is further stabilised by two hydrogen bonds between the main-chain nitrogen of Val345 and a carbonyl oxygen of Val347 of respective subunits. (D) Dimer interface of the TgMORN1(7–15) V-shaped dimer. Cys305 and Asp308 incorporate a structural Zn2+ ion, which stabilises the somewhat smaller dimerisation interface of this protein. Although its dimerisation interface is very similar to that of PfMORN1(7–15), it lacks the aromatic core of PfMORN1(7–15). The latter is replaced by a series of unique aromatic stacking interactions at the protein´s vertex, these being contributed by a pair of Phe350 residues, sandwiched between a pair of His333 residues. (E) Kratky plots derived from SAXS analysis of TbMORN1(7–15), TgMORN1(7–15), PfMORN1(7–15), and TbMORN1(2–15). The shape of the plots suggests an elongated shape of the dimers in solution. (TIF) [file pone.0242677.s012.tif]

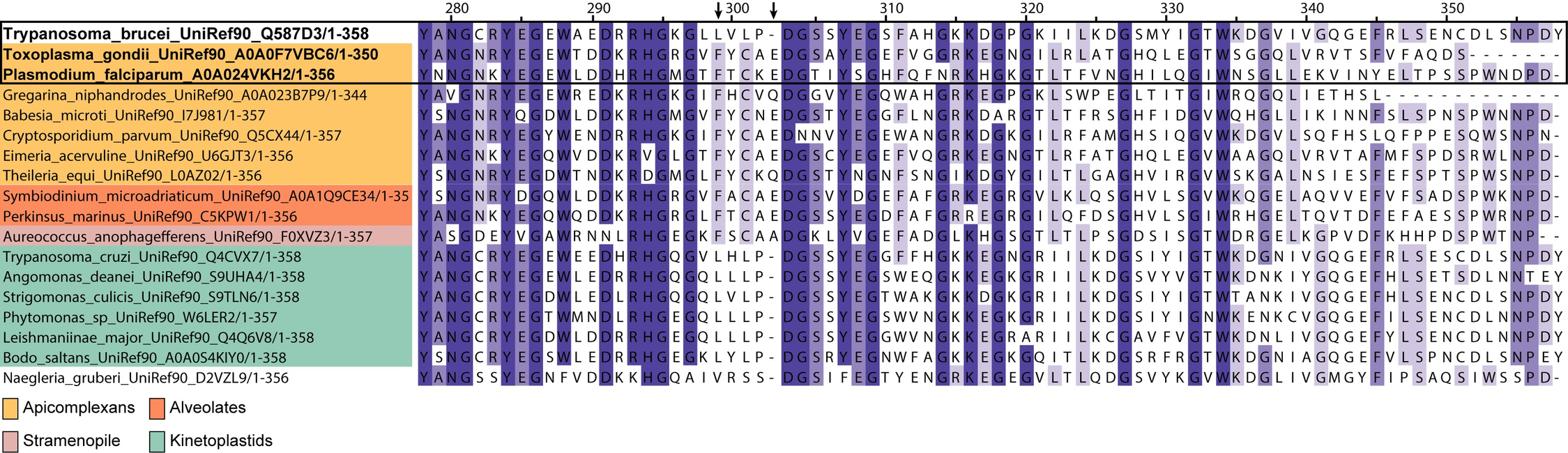

Supplement: S13 Fig — Amino acid sequence alignment of the C-termini of TbMORN1, TgMORN1, PfMORN1, and fifteen other MORN repeat-containing proteins from related taxa. Amino acid numbers are given according to the TbMORN1 protein, and the three proteins with experimentally-determined high-resolution structures are shown in bold within the black box. Essential for formation of a V-shaped dimer are a coordinating Cys residues and an anion-π interaction pair. In TbMORN1, the coordinating Cys residue has been substituted for Leu (Leu 301). Similarly, the Phe and Glu residues of the anion-π interaction pair (indicated with black arrows) have been substituted for Leu (Leu299) and are not present (deletion after Pro302) respectively. This supports the conclusion that TbMORN1 exists only in the extended form, while the apicomplexan proteins and those from related clades are probably capable of adopting both extended and V-shaped conformations. (TIF) [file pone.0242677.s013.tif]

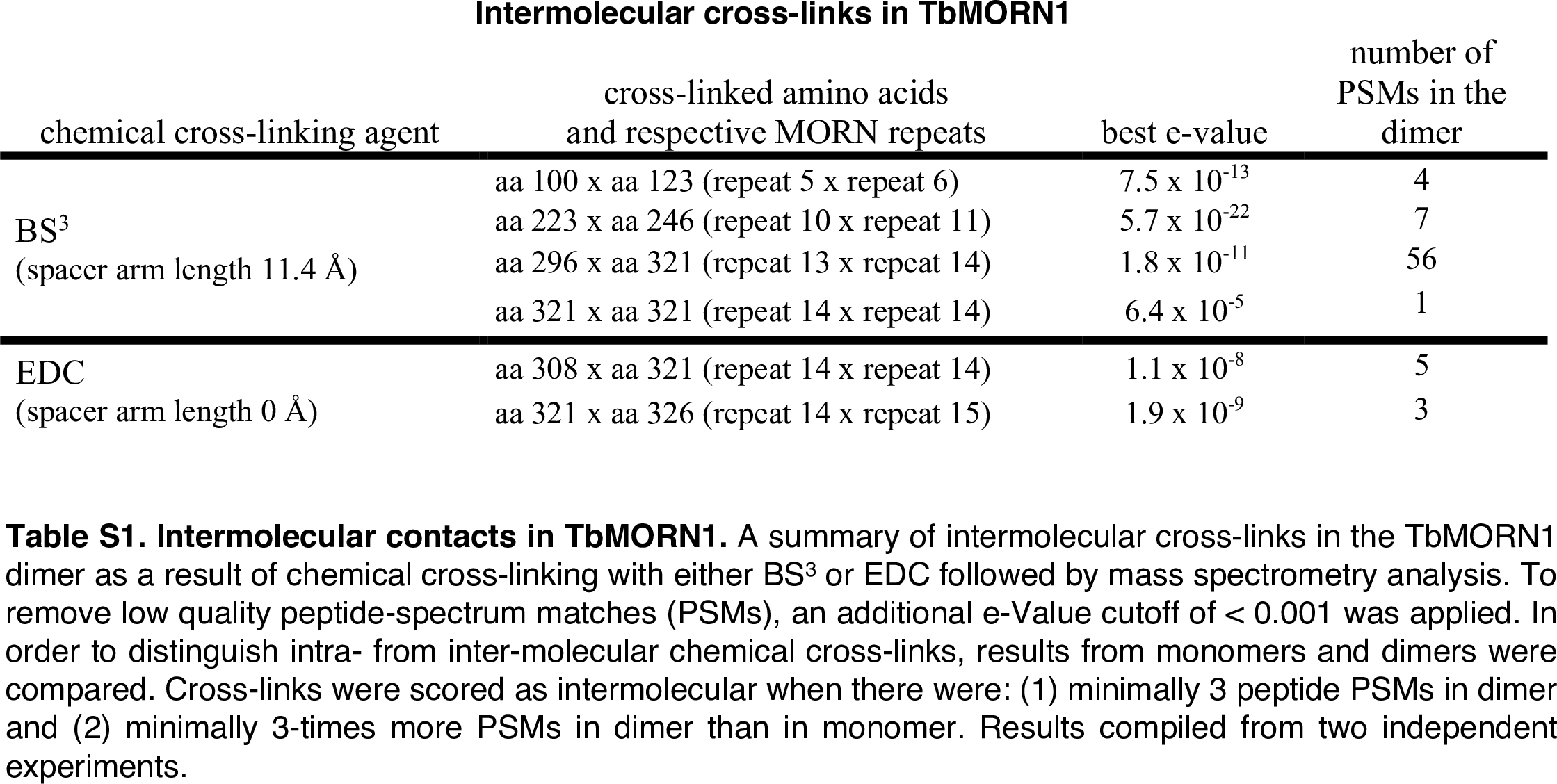

Supplement: S1 Table — (TIF) [file pone.0242677.s014.tif]

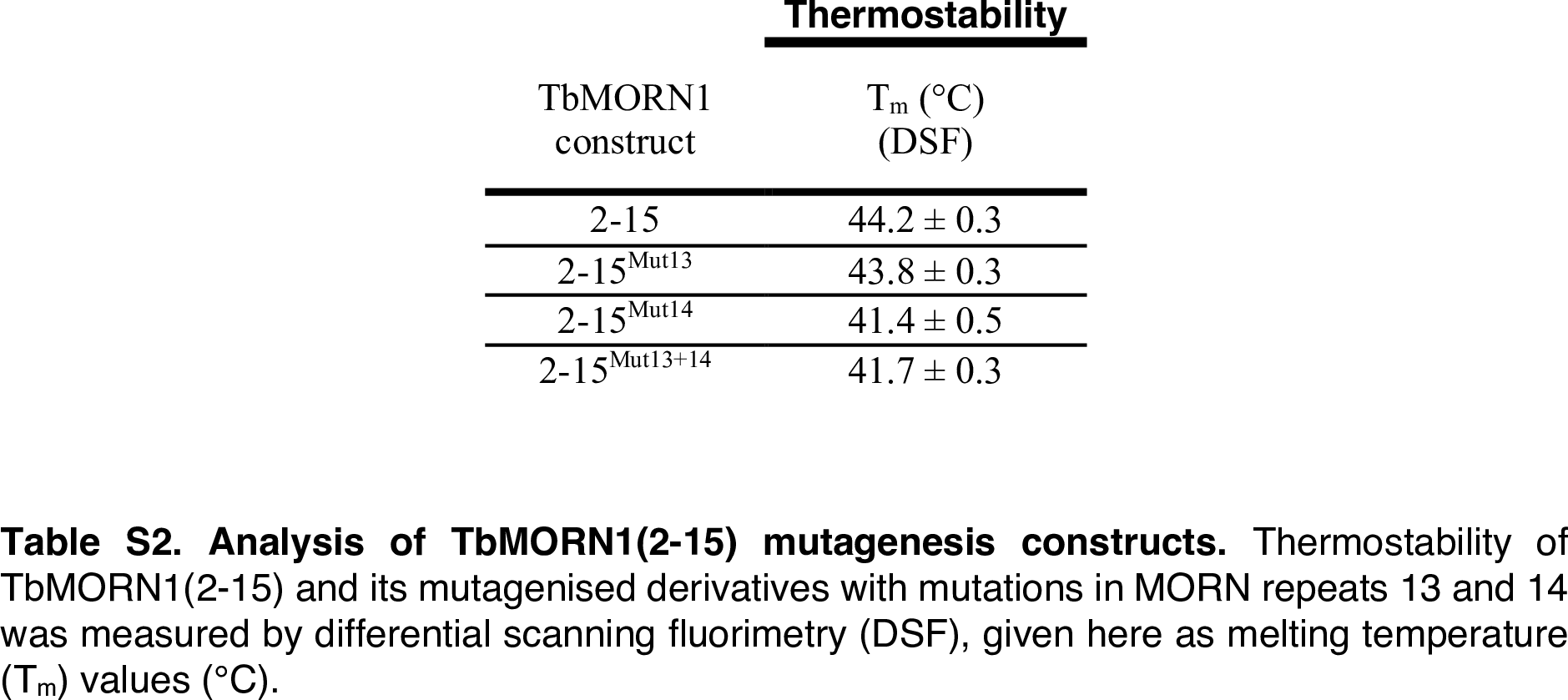

Supplement: S2 Table — (TIF) [file pone.0242677.s015.tif]

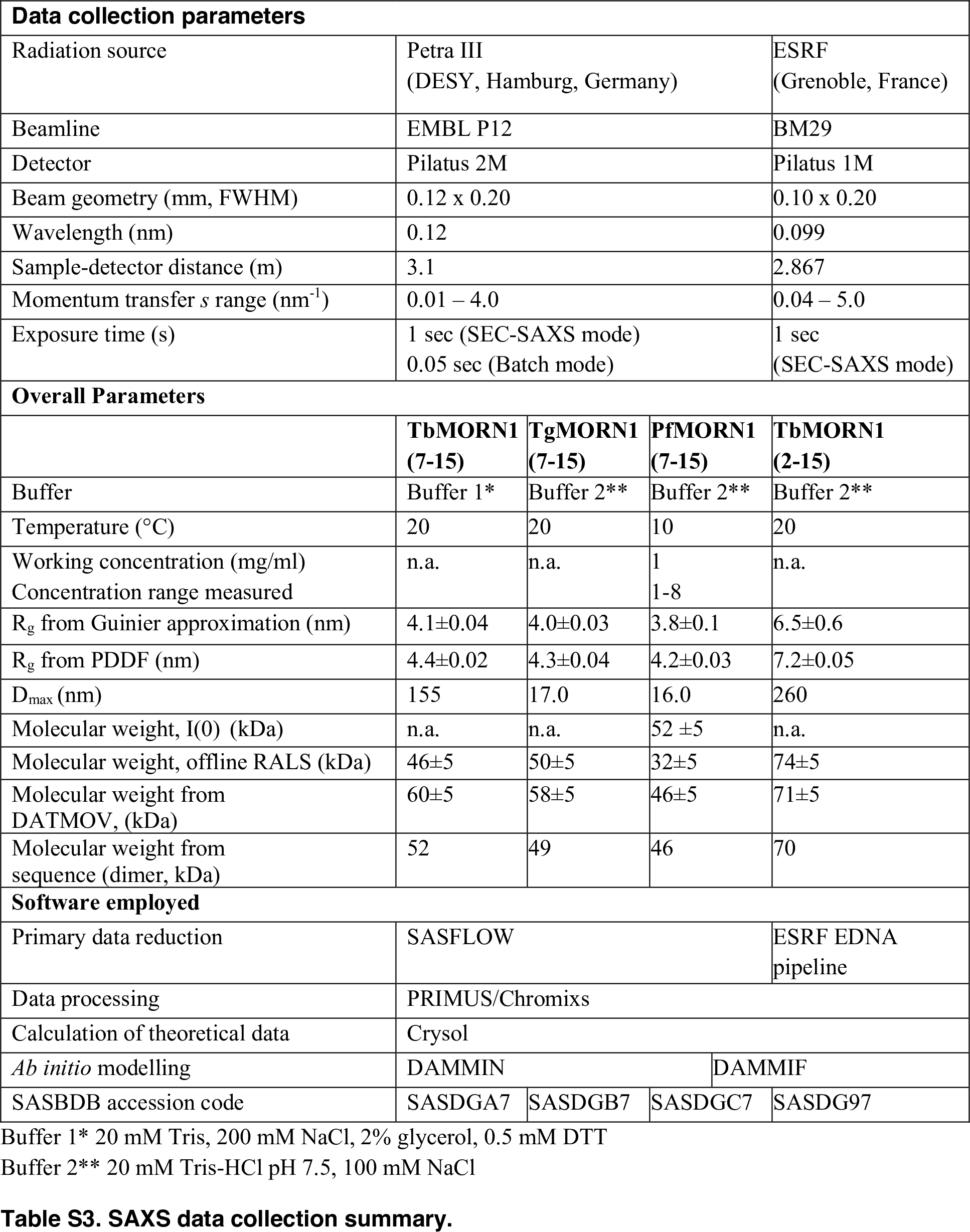

Supplement: S3 Table — (TIF) [file pone.0242677.s016.tif]
