# Supplementary material for: Structures of three MORN repeat proteins and a re-evaluation of the proposed lipid-binding properties of MORN repeats
Source: PLoS One. 2020 Dec 9;15(12):e0242677. doi: 10.1371/journal.pone.0242677 (PMC7725318; doi:10.1371/journal.pone.0242677)
Supplement: S1 Raw images — (PDF) [file pone.0242677.s018.pdf]

Raw data for Fig 3C, Fig 3G

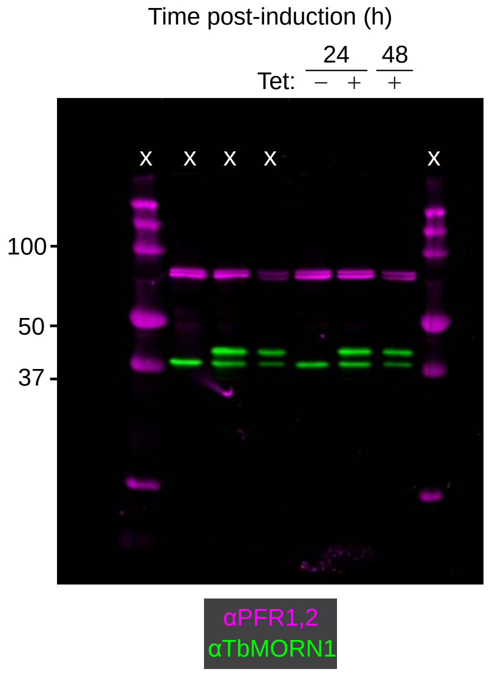

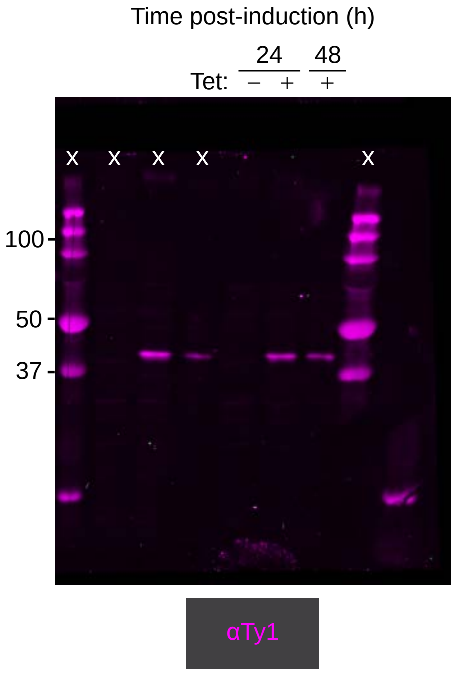

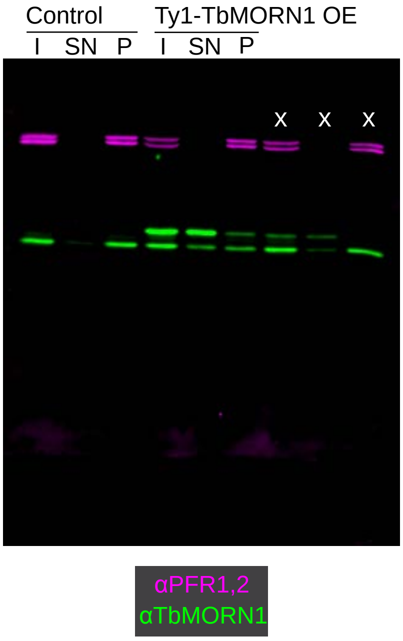

Images obtained using Odyssey CLx (Li-Cor)

Raw data for Fig 4B

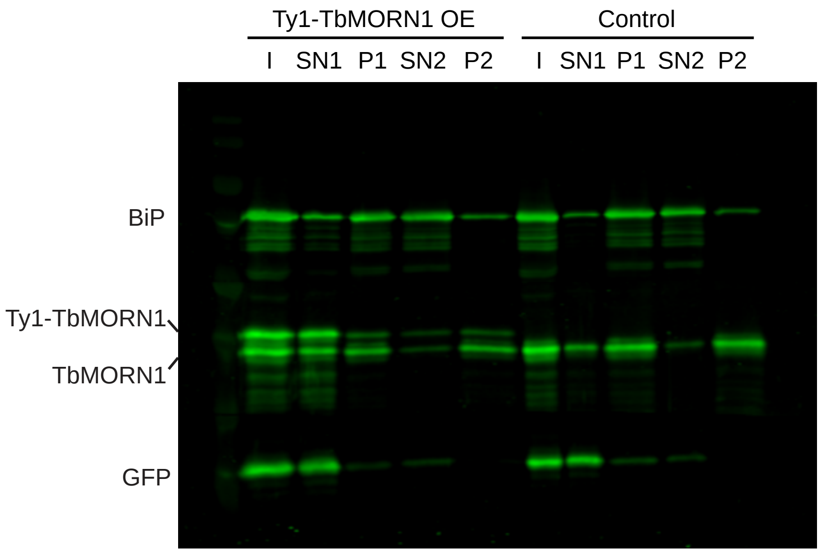

Images obtained using Odyssey CLx (Li-Cor)

Raw data for Fig S1A

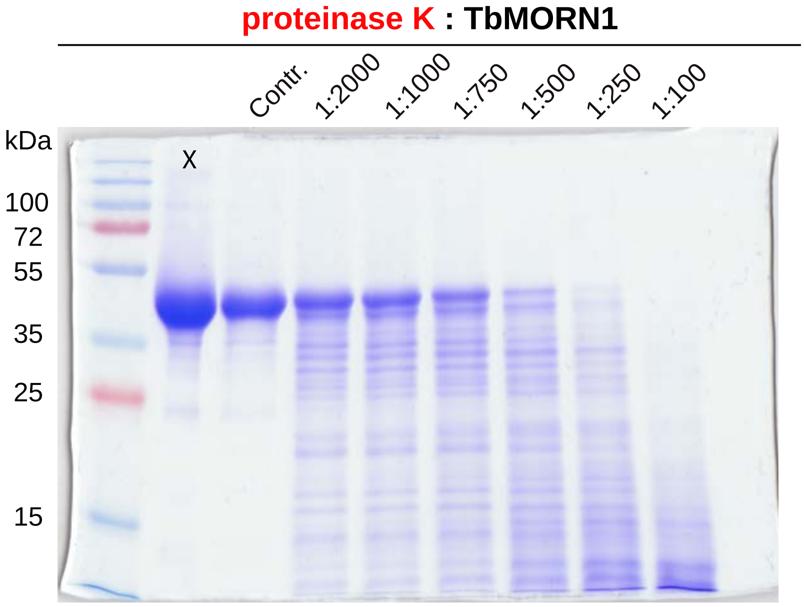

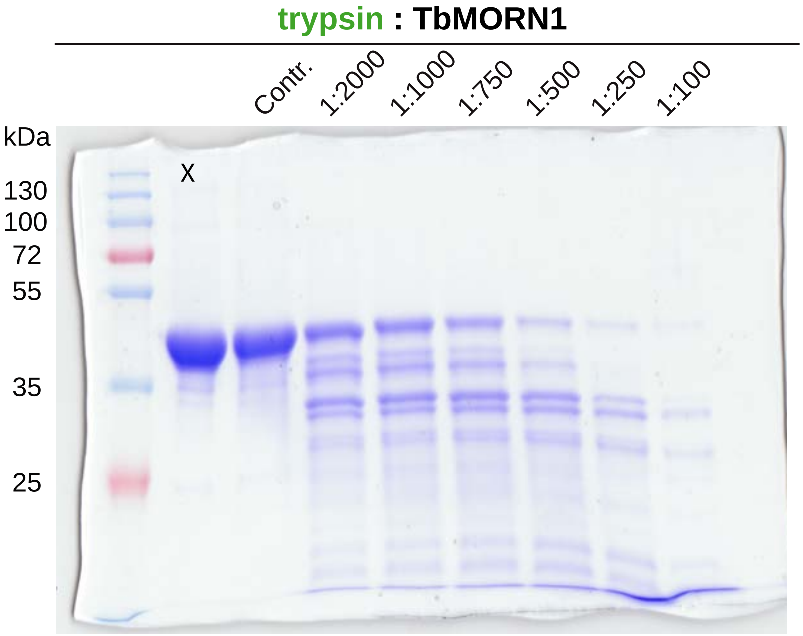

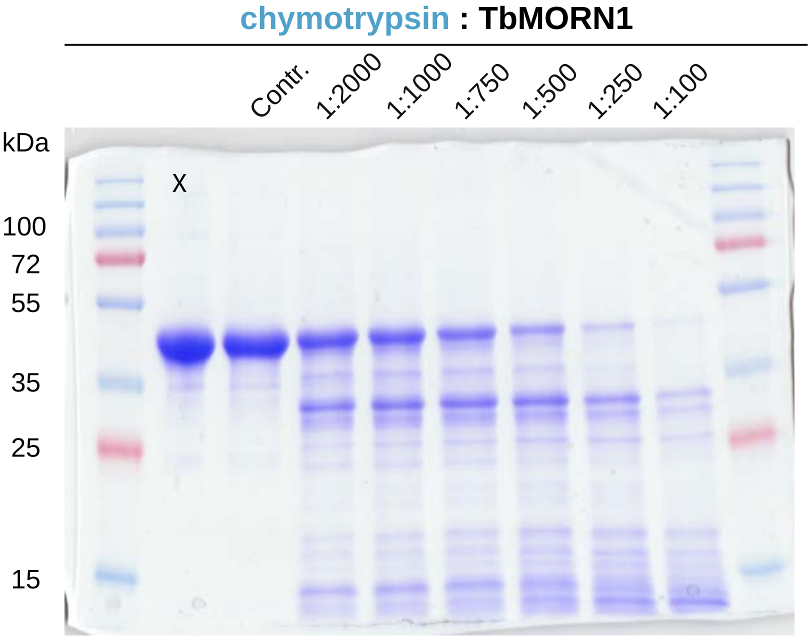

Images obtained using a digital camera

Raw data for Fig S1C

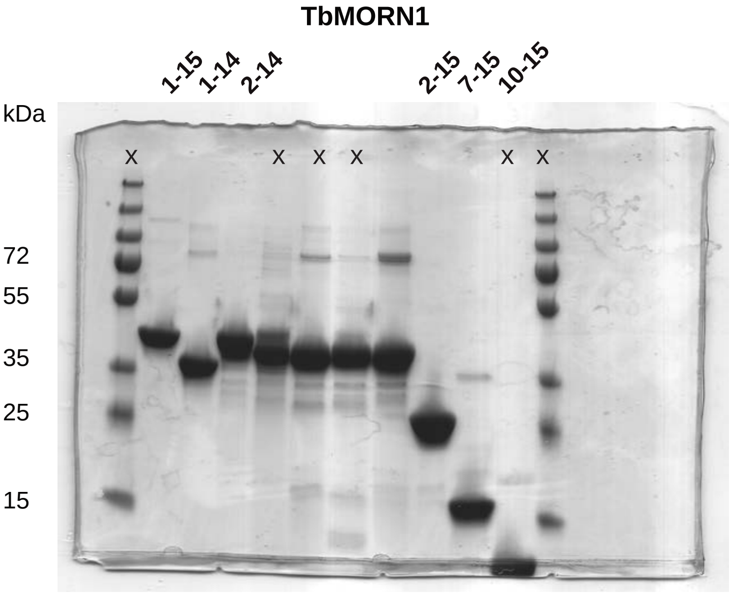

Images obtained using a digital camera

Raw data for Fig S2D

TbMORN1(2-15)

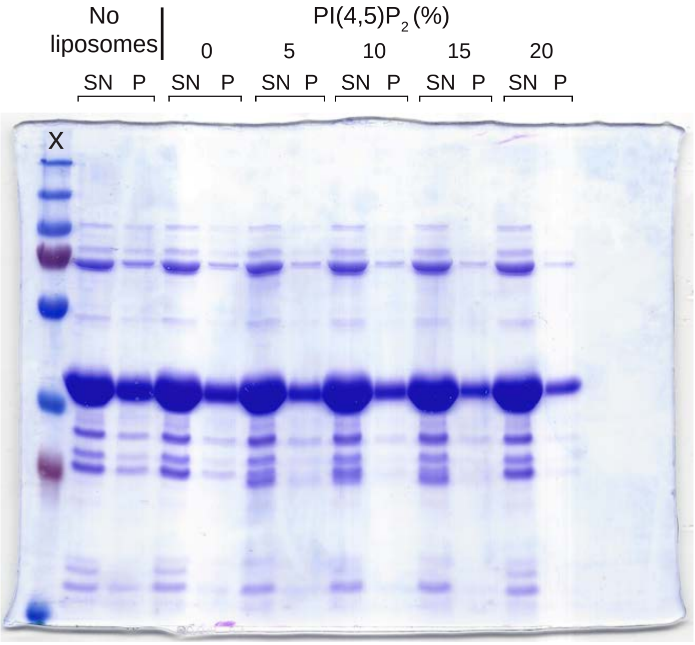

positive control: 10 μM Doc2B

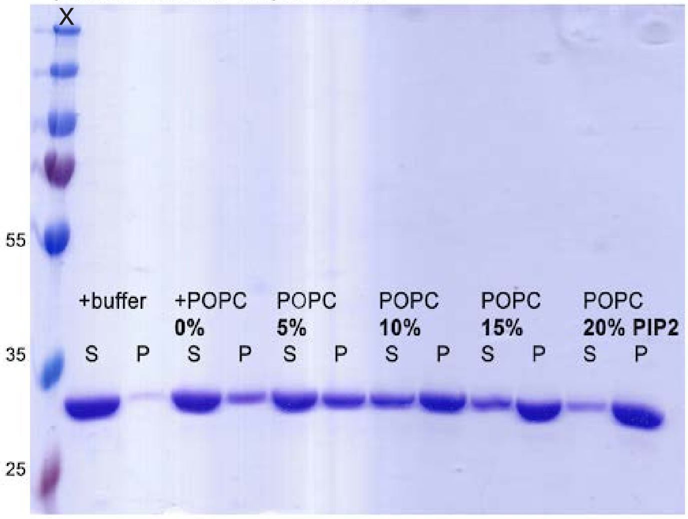

Images obtained using a digital camera

Raw data for Fig S3

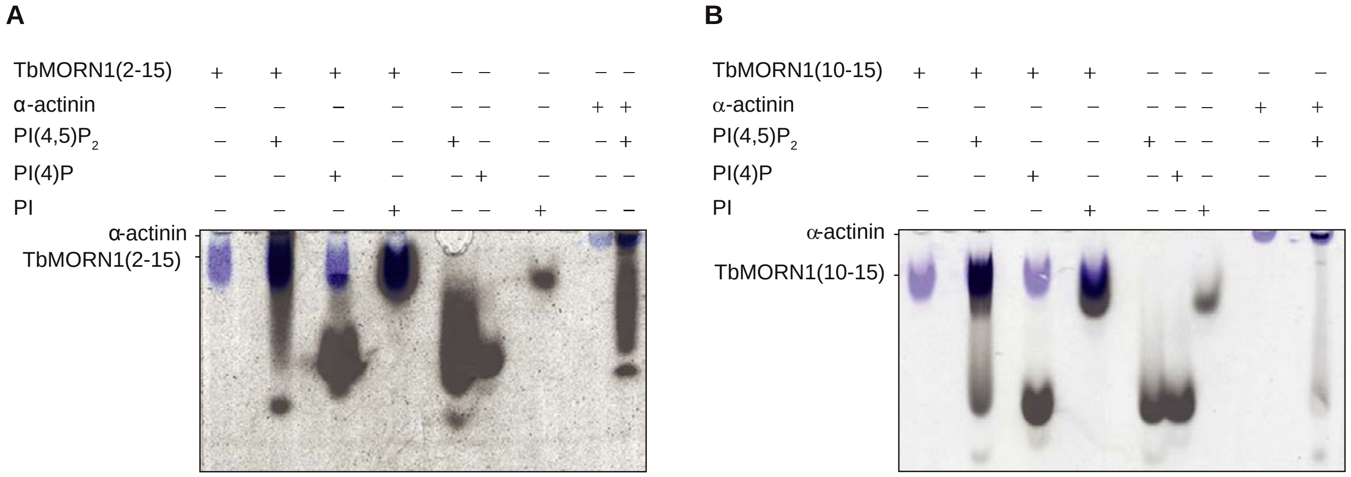

Images obtained using a digital camera

Raw data for Fig S4A

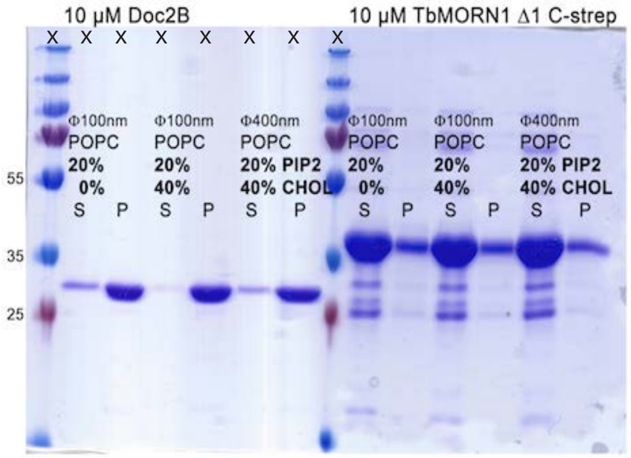

Images obtained using a digital camera

Raw data for Fig 6ABC

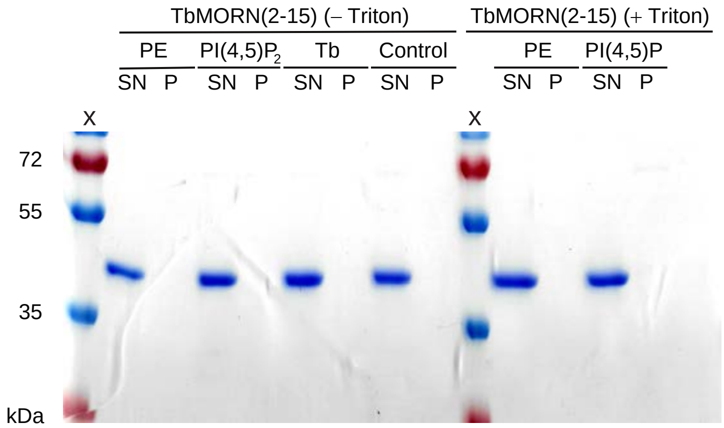

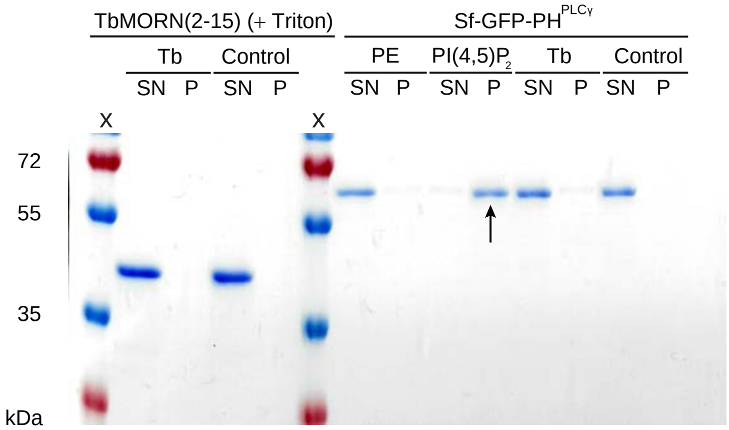

Images obtained using a digital camera

Raw data for Fig S7A

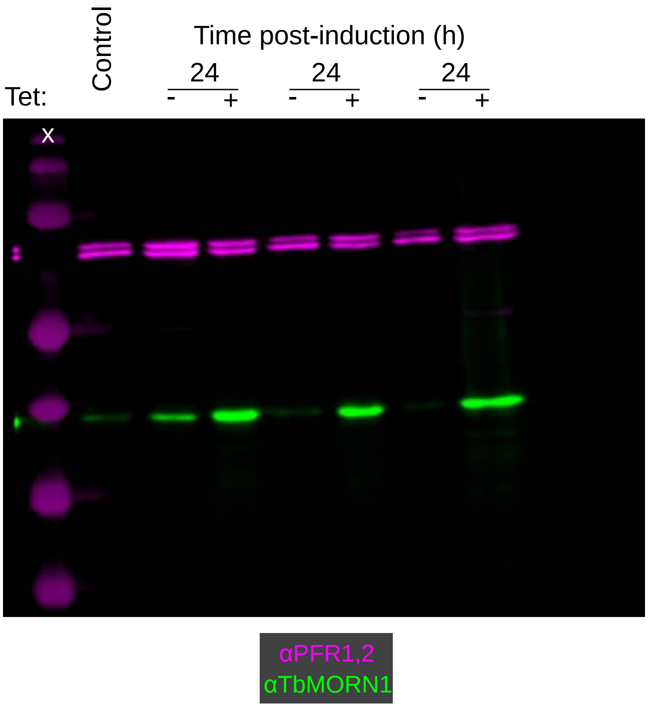

Images obtained using Odyssey CLx (Li-Cor)

Raw data for Fig S8B

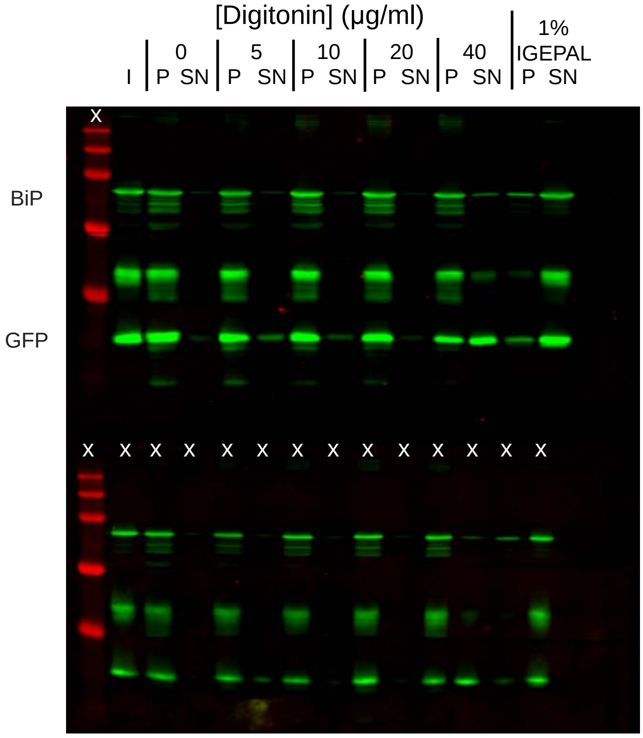

Images obtained using Odyssey CLx (Li-Cor)

Raw data for Fig S8E

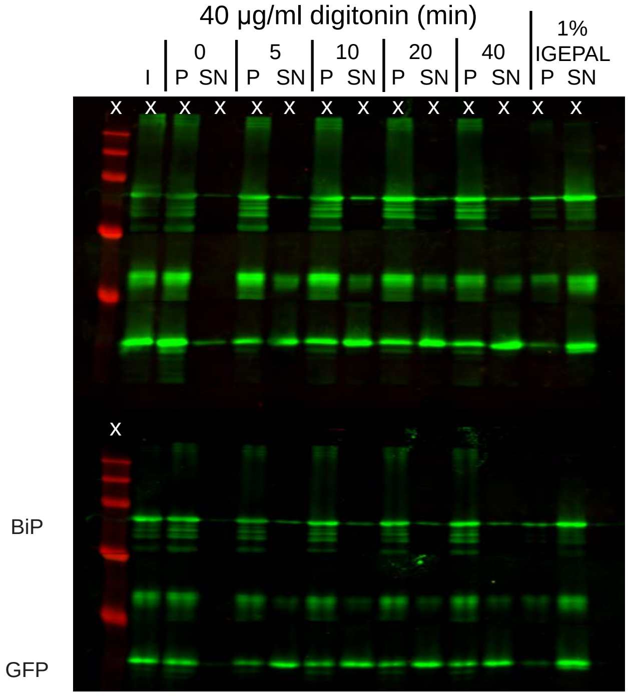

Images obtained using Odyssey CLx (Li-Cor)
