# Supplementary material for: Structures of three MORN repeat proteins and a re-evaluation of the proposed lipid-binding properties of MORN repeats
Source: PLoS One. 2020 Dec 9;15(12):e0242677. doi: 10.1371/journal.pone.0242677 (PMC7725318; doi:10.1371/journal.pone.0242677)
Supplement: S4 File — (PDF) [file pone.0242677.s022.pdf]

Full wwPDB X-ray Structure Validation Report (i)

Oct 18, 2019 – 08:50 am BST

PDB ID	:	6T68
Title	:	Crystal structure of Trypanosoma brucei Morn1
Deposited on	:	2019-10-17
Resolution	:	2.54 Å(reported)

This is a Full wwPDB X-ray Structure Validation Report.

This report is produced by the wwPDB biocuration pipeline after annotation of the structure.

We welcome your comments at *validation@mail.wwpdb.org*
A user guide is available at
https://www.wwpdb.org/validation/2017/XrayValidationReportHelp
with specific help available everywhere you see the (i) symbol.

CONFIDENTIAL VALIDATION REPORT

The following versions of software and data (see references (i)) were used in the production of this report:

MolProbity	:	4.02b-467
Xtriage (Phenix)	:	1.13
EDS	:	2.5
Percentile statistics	:	20171227.v01 (using entries in the PDB archive December 27th 2017)
Refmac	:	5.8.0158
CCP4	:	7.0 (Gargrove)
Ideal geometry (proteins)	:	Engh & Huber (2001)
Ideal geometry (DNA, RNA)	:	Parkinson et al. (1996)
Validation Pipeline (wwPDB-VP)	:	2.5

1 Overall quality at a glance (i)

The following experimental techniques were used to determine the structure:
X-RAY DIFFRACTION

The reported resolution of this entry is 2.54 Å.

Percentile scores (ranging between 0-100) for global validation metrics of the entry are shown in the following graphic. The table shows the number of entries on which the scores are based.

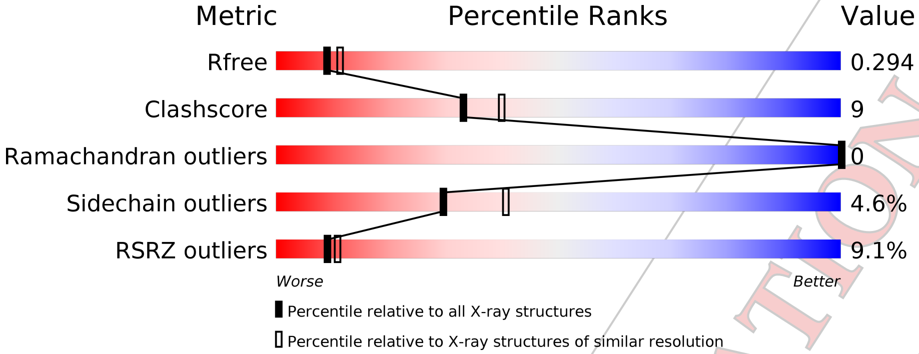

Metric	Whole archive (#Entries)	Similar resolution (#Entries, resolution range(Å))
R_{free}	111664	1053 (2.56-2.52)
Clashscore	122126	1098 (2.56-2.52)
Ramachandran outliers	120053	1088 (2.56-2.52)
Sidechain outliers	120020	1088 (2.56-2.52)
RSRZ outliers	108989	1043 (2.56-2.52)

The table below summarises the geometric issues observed across the polymeric chains and their fit to the electron density. The red, orange, yellow and green segments on the lower bar indicate the fraction of residues that contain outliers for >=3, 2, 1 and 0 types of geometric quality criteria. A grey segment represents the fraction of residues that are not modelled. The numeric value for each fraction is indicated below the corresponding segment, with a dot representing fractions <=5% The upper red bar (where present) indicates the fraction of residues that have poor fit to the electron density. The numeric value is given above the bar.

Mol	Chain	Length	Quality of chain
1	A	216	6%<br>74% 18% 8%
1	B	216	11%<br>68% 21% • 9%

CONFIDENTIAL VALIDATION REPORT

2 Entry composition (i)

There are 3 unique types of molecules in this entry. The entry contains 3197 atoms, of which 0 are hydrogens and 0 are deuteriums.

In the tables below, the ZeroOcc column contains the number of atoms modelled with zero occupancy, the AltConf column contains the number of residues with at least one atom in alternate conformation and the Trace column contains the number of residues modelled with at most 2 atoms.

- Molecule 1 is a protein called MORN repeat-containing protein 1.

Mol	Chain	Residues	Atoms	ZeroOcc	AltConf	Trace
1	A	198	Total 1592, C 1009, N 276, O 303, S 4	0	0	0
1	B	197	Total 1584, C 1004, N 275, O 300, S 5	0	0	0

- Molecule 2 is CHLORIDE ION (three-letter code: CL) (formula: Cl).

Mol	Chain	Residues	Atoms	ZeroOcc	AltConf
2	B	3	Total 3, Cl 3	0	0
2	A	2	Total 2, Cl 2	0	0

- Molecule 3 is water.

Mol	Chain	Residues	Atoms	ZeroOcc	AltConf
3	A	7	Total 7, O 7	0	0
3	B	9	Total 9, O 9	0	0

CONFIDENTIAL VALIDATION REPORT

3 Residue-property plots ⓘ

These plots are drawn for all protein, RNA and DNA chains in the entry. The first graphic for a chain summarises the proportions of the various outlier classes displayed in the second graphic. The second graphic shows the sequence view annotated by issues in geometryand electron density. Residues are color-coded according to the number of geometric quality criteria for which they contain at least one outlier: green = 0, yellow = 1, orange = 2 and red = 3 or more. A red dot above a residue indicates a poor fit to the electron density (RSRZ > 2). Stretches of 2 or more consecutive residues without any outlier are shown as a green connector. Residues present in the sample, but not in the model, are shown in grey.

- Molecule 1: MORN repeat-containing protein 1

Chain A:

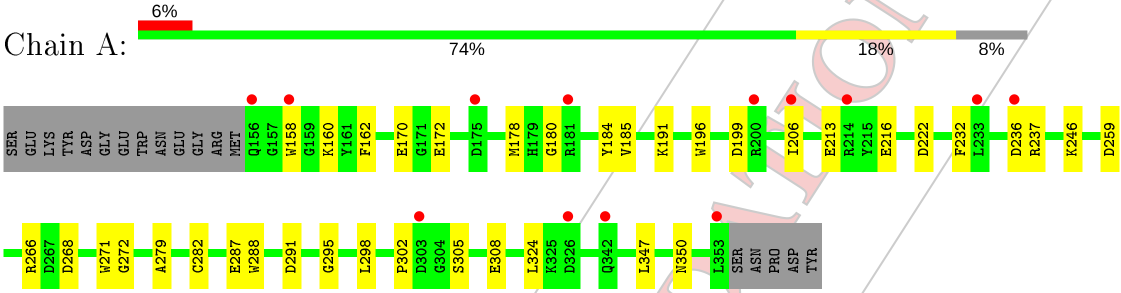

- Molecule 1: MORN repeat-containing protein 1

Chain B:

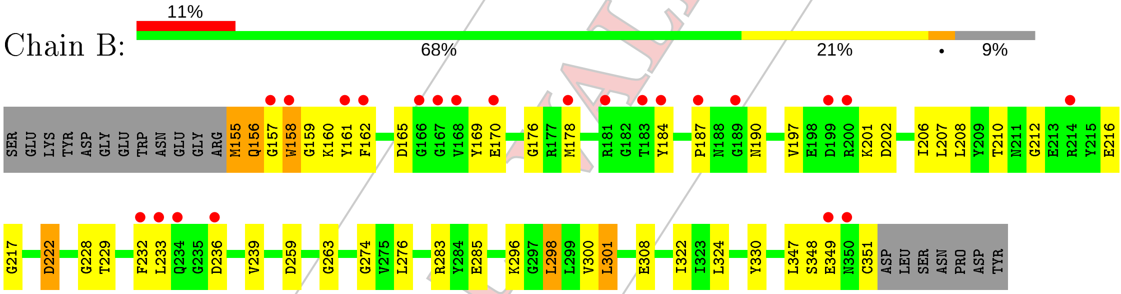

CONFIDENTIAL VALIDATION REPORT

4 Data and refinement statistics (i)

Property	Value	Source
Space group	C 1 2 1	Depositor
Cell constants<br>a, b, c, α, β, γ	192.88Å 49.74Å 41.98Å<br>90.00° 97.54° 90.00°	Depositor
Resolution (Å)	48.14 – 2.54<br>48.14 – 2.53	Depositor<br>EDS
% Data completeness<br>(in resolution range)	92.7 (48.14-2.54)<br>87.1 (48.14-2.53)	Depositor<br>EDS
R_{merge}	0.12	Depositor
R_{sym}	(Not available)	Depositor
$< I/\sigma(I) >$ [1]	0.34 (at 2.54Å)	Xtriage
Refinement program	PHENIX 1.17_3644, PHENIX 1.17_3644	Depositor
R, R_{free}	0.234 , 0.293<br>0.234 , 0.294	Depositor<br>DCC
R_{free} test set	610 reflections (4.91%)	wwPDB-VP
Wilson B-factor (Å^2)	48.9	Xtriage
Anisotropy	0.483	Xtriage
Bulk solvent k_{sol}(e/Å^3), B_{sol}(Å^2)	0.35 , 66.5	EDS
L-test for twinning[2]	$< \|L\| >$ = 0.49, $< L^2 >$ = 0.33	Xtriage
Estimated twinning fraction	No twinning to report.	Xtriage
F_o,F_c correlation	0.93	EDS
Total number of atoms	3197	wwPDB-VP
Average B, all atoms (Å^2)	66.0	wwPDB-VP

Xtriage's analysis on translational NCS is as follows: *The largest off-origin peak in the Patterson function is 10.45% of the height of the origin peak. No significant pseudotranslation is detected.*

CONFIDENTIAL VALIDATION REPORT

[1] Intensities estimated from amplitudes.
[2] Theoretical values of $< |L| >$, $< L^2 >$ for acentric reflections are 0.5, 0.333 respectively for untwinned datasets, and 0.375, 0.2 for perfectly twinned datasets.

5 Model quality (i)

5.1 Standard geometry (i)

Bond lengths and bond angles in the following residue types are not validated in this section: CL

The Z score for a bond length (or angle) is the number of standard deviations the observed value is removed from the expected value. A bond length (or angle) with $|Z| > 5$ is considered an outlier worth inspection. RMSZ is the root-mean-square of all Z scores of the bond lengths (or angles).

Mol	Chain	Bond lengths		Bond angles	
		RMSZ	$\#|Z| > 5$	RMSZ	$\#|Z| > 5$
1	A	0.31	0/1643	0.49	0/2215
1	B	0.30	0/1635	0.51	0/2203
All	All	0.30	0/3278	0.50	0/4418

There are no bond length outliers.

There are no bond angle outliers.

There are no chirality outliers.

There are no planarity outliers.

5.2 Too-close contacts (i)

In the following table, the Non-H and H(model) columns list the number of non-hydrogen atoms and hydrogen atoms in the chain respectively. The H(added) column lists the number of hydrogen atoms added and optimized by MolProbity. The Clashes column lists the number of clashes within the asymmetric unit, whereas Symm-Clashes lists symmetry related clashes.

Mol	Chain	Non-H	H(model)	H(added)	Clashes	Symm-Clashes
1	A	1592	0	1436	21	1
1	B	1584	0	1430	38	1
2	A	2	0	0	0	0
2	B	3	0	0	0	0
3	A	7	0	0	0	0
3	B	9	0	0	0	0
All	All	3197	0	2866	56	1

The all-atom clashscore is defined as the number of clashes found per 1000 atoms (including hydrogen atoms). The all-atom clashscore for this structure is 9.

All (56) close contacts within the same asymmetric unit are listed below, sorted by their clash

CONFIDENTIAL VALIDATION REPORT

magnitude.

Atom-1	Atom-2	Interatomic distance (Å)	Clash overlap (Å)
1:B:347:LEU:HD23	1:B:349:GLU:H	1.06	1.13
1:B:347:LEU:CD2	1:B:349:GLU:H	1.67	1.05
1:B:347:LEU:HD23	1:B:349:GLU:N	1.83	0.92
1:B:347:LEU:HD22	1:B:349:GLU:O	1.75	0.86
1:A:347:LEU:HD21	1:B:322:ILE:HD11	1.61	0.81
1:B:165:ASP:HB2	1:B:187:PRO:HG3	1.68	0.74
1:B:347:LEU:HD23	1:B:348:SER:N	2.04	0.71
1:A:213:GLU:HG2	1:A:232:PHE:HA	1.77	0.66
1:B:206:ILE:HG12	1:B:216:GLU:HG3	1.79	0.64
1:B:259:ASP:HB3	1:B:276:LEU:HD11	1.81	0.63
1:B:283:ARG:NH1	1:B:285:GLU:OE1	2.31	0.63
1:A:160:LYS:HG3	1:A:170:GLU:HB3	1.81	0.62
1:A:350:ASN:HA	1:B:301:LEU:HD22	1.82	0.61
1:B:347:LEU:CD2	1:B:349:GLU:N	2.51	0.60
1:A:259:ASP:OD1	1:A:279:ALA:N	2.34	0.60
1:B:155:MET:HB2	1:B:176:GLY:H	1.69	0.58
1:A:298:LEU:HD13	1:A:308:GLU:HB3	1.87	0.57
1:B:212:GLY:O	1:B:233:LEU:HD23	2.08	0.54
1:B:169:TYR:CE2	1:B:178:MET:HG2	2.43	0.53
1:B:322:ILE:HG23	1:B:330:TYR:HB3	1.90	0.52
1:B:232:PHE:CD2	1:B:236:ASP:HB2	2.45	0.52
1:A:160:LYS:HD2	1:A:162:PHE:CE1	2.45	0.52
1:A:178:MET:N	1:A:199:ASP:OD1	2.36	0.51
1:A:282:CYS:SG	1:A:302:PRO:HD3	2.50	0.51
1:B:232:PHE:HB2	1:B:236:ASP:HB2	1.92	0.50
1:B:283:ARG:HB3	1:B:300:VAL:HG23	1.93	0.50
1:B:229:THR:HG23	1:B:239:VAL:HG22	1.94	0.49
1:B:156:GLN:HG2	1:B:157:GLY:N	2.28	0.49
1:B:160:LYS:HE2	1:B:170:GLU:HB3	1.95	0.48
1:A:185:VAL:HA	1:A:191:LYS:HA	1.96	0.47
1:B:296:LYS:H	1:B:296:LYS:HD2	1.81	0.46
1:A:246:LYS:HG2	1:A:268:ASP:HB2	1.98	0.46
1:B:190:ASN:HB3	1:B:207:LEU:HD21	1.98	0.46
1:A:206:ILE:HG23	1:A:216:GLU:HG2	1.97	0.45
1:B:190:ASN:OD1	1:B:210:THR:HG23	2.16	0.45
1:A:158:TRP:CD1	1:A:172:GLU:HG2	2.51	0.45
1:B:298:LEU:HD12	1:B:308:GLU:HB3	1.99	0.45
1:A:180:GLY:O	1:A:196:TRP:N	2.50	0.45
1:A:350:ASN:HA	1:B:301:LEU:CD2	2.47	0.44
1:B:197:VAL:HB	1:B:202:ASP:HB3	1.98	0.44
1:A:272:GLY:O	1:A:288:TRP:N	2.50	0.44

Continued on next page...

CONFIDENTIAL VALIDATION REPORT

Continued from previous page...

Atom-1	Atom-2	Interatomic distance (Å)	Clash overlap (Å)
1:A:305:SER:HB3	1:A:324:LEU:HA	1.99	0.44
1:B:322:ILE:CG2	1:B:330:TYR:HB3	2.47	0.44
1:B:158:TRP:HB3	1:B:159:GLY:H	1.63	0.44
1:B:155:MET:HB2	1:B:176:GLY:N	2.32	0.43
1:B:162:PHE:CE2	1:B:178:MET:SD	3.12	0.43
1:B:232:PHE:CG	1:B:236:ASP:HB2	2.54	0.43
1:B:201:LYS:HD2	1:B:222:ASP:OD1	2.20	0.42
1:B:169:TYR:CD2	1:B:178:MET:HG2	2.54	0.42
1:A:236:ASP:OD2	1:A:236:ASP:N	2.52	0.42
1:A:158:TRP:N	1:A:158:TRP:CD1	2.86	0.42
1:B:263:GLY:HA3	1:B:274:GLY:HA2	2.02	0.41
1:B:324:LEU:HD22	1:B:324:LEU:N	2.35	0.41
1:B:217:GLY:HA3	1:B:228:GLY:HA2	2.02	0.41
1:A:266:ARG:HB2	1:A:271:TRP:HB3	2.01	0.41
1:A:287:GLU:HG3	1:A:295:GLY:HA3	2.02	0.41

All (1) symmetry-related close contacts are listed below. The label for Atom-2 includes the symmetry operator and encoded unit-cell translations to be applied.

Atom-1	Atom-2	Interatomic distance (Å)	Clash overlap (Å)
1:A:287:GLU:OE2	1:B:283:ARG:NH2[4_549]	2.10	0.10

5.3 Torsion angles (i)

5.3.1 Protein backbone (i)

In the following table, the Percentiles column shows the percent Ramachandran outliers of the chain as a percentile score with respect to all X-ray entries followed by that with respect to entries of similar resolution.

The Analysed column shows the number of residues for which the backbone conformation was analysed, and the total number of residues.

Mol	Chain	Analysed	Favoured	Allowed	Outliers	Percentiles	
1	A	196/216 (91%)	191 (97%)	5 (3%)	0	100	100
1	B	195/216 (90%)	185 (95%)	10 (5%)	0	100	100
All	All	391/432 (90%)	376 (96%)	15 (4%)	0	100	100

There are no Ramachandran outliers to report.

CONFIDENTIAL VALIDATION REPORT

5.3.2 Protein sidechains (i)

In the following table, the Percentiles column shows the percent sidechain outliers of the chain as a percentile score with respect to all X-ray entries followed by that with respect to entries of similar resolution.

The Analysed column shows the number of residues for which the sidechain conformation was analysed, and the total number of residues.

Mol	Chain	Analysed	Rotameric	Outliers	Percentiles	
1	A	153/169 (90%)	149 (97%)	4 (3%)	49	65
1	B	152/169 (90%)	142 (93%)	10 (7%)	18	25
All	All	305/338 (90%)	291 (95%)	14 (5%)	29	41

All (14) residues with a non-rotameric sidechain are listed below:

Mol	Chain	Res	Type
1	A	184	TYR
1	A	222	ASP
1	A	237	ARG
1	A	291	ASP
1	B	155	MET
1	B	156	GLN
1	B	158	TRP
1	B	161	TYR
1	B	184	TYR
1	B	208	LEU
1	B	222	ASP
1	B	298	LEU
1	B	301	LEU
1	B	351	CYS

Some sidechains can be flipped to improve hydrogen bonding and reduce clashes. There are no such sidechains identified.

5.3.3 RNA (i)

There are no RNA molecules in this entry.

5.4 Non-standard residues in protein, DNA, RNA chains (i)

There are no non-standard protein/DNA/RNA residues in this entry.

CONFIDENTIAL VALIDATION REPORT

5.5 Carbohydrates (i)

There are no carbohydrates in this entry.

5.6 Ligand geometry (i)

Of 5 ligands modelled in this entry, 5 are monoatomic - leaving 0 for Mogul analysis.

There are no bond length outliers.

There are no bond angle outliers.

There are no chirality outliers.

There are no torsion outliers.

There are no ring outliers.

No monomer is involved in short contacts.

5.7 Other polymers (i)

There are no such residues in this entry.

5.8 Polymer linkage issues (i)

There are no chain breaks in this entry.

CONFIDENTIAL VALIDATION REPORT

6 Fit of model and data ⓘ

6.1 Protein, DNA and RNA chains ⓘ

In the following table, the column labelled '#RSRZ> 2' contains the number (and percentage) of RSRZ outliers, followed by percent RSRZ outliers for the chain as percentile scores relative to all X-ray entries and entries of similar resolution. The OWAB column contains the minimum, median, 95th percentile and maximum values of the occupancy-weighted average B-factor per residue. The column labelled 'Q< 0.9' lists the number of (and percentage) of residues with an average occupancy less than 0.9.

Mol	Chain	Analysed	<RSRZ>	#RSRZ>2			OWAB(Å^2)	Q<0.9
1	A	198/216 (91%)	0.63	13 (6%)	18	21	34, 62, 101, 120	0
1	B	197/216 (91%)	0.77	23 (11%)	4	6	38, 63, 104, 124	0
All	All	395/432 (91%)	0.70	36 (9%)	9	11	34, 63, 103, 124	0

All (36) RSRZ outliers are listed below:

Mol	Chain	Res	Type	RSRZ
1	A	353	LEU	5.3
1	B	166	GLY	5.3
1	A	158	TRP	5.0
1	A	156	GLN	4.6
1	B	157	GLY	4.4
1	A	233	LEU	4.2
1	B	162	PHE	3.7
1	B	158	TRP	3.6
1	B	200	ARG	3.5
1	A	206	ILE	3.4
1	B	167	GLY	3.4
1	B	350	ASN	3.3
1	B	161	TYR	3.1
1	A	175	ASP	3.0
1	B	184	TYR	3.0
1	B	178	MET	2.9
1	B	233	LEU	2.8
1	B	234	GLN	2.8
1	B	232	PHE	2.7
1	B	349	GLU	2.6
1	B	183	THR	2.6
1	A	181	ARG	2.6
1	A	200	ARG	2.5
1	B	187	PRO	2.5

Continued on next page...

CONFIDENTIAL VALIDATION REPORT

Continued from previous page...

Mol	Chain	Res	Type	RSRZ
1	B	170	GLU	2.5
1	B	236	ASP	2.4
1	B	189	GLY	2.4
1	A	303	ASP	2.4
1	A	214	ARG	2.4
1	B	168	VAL	2.3
1	B	181	ARG	2.3
1	B	214	ARG	2.2
1	A	236	ASP	2.2
1	A	326	ASP	2.1
1	A	342	GLN	2.0
1	B	199	ASP	2.0

6.2 Non-standard residues in protein, DNA, RNA chains (i)

There are no non-standard protein/DNA/RNA residues in this entry.

6.3 Carbohydrates (i)

There are no carbohydrates in this entry.

6.4 Ligands (i)

In the following table, the Atoms column lists the number of modelled atoms in the group and the number defined in the chemical component dictionary. The B-factors column lists the minimum, median, 95th percentile and maximum values of B factors of atoms in the group. The column labelled '$Q< 0.9$' lists the number of atoms with occupancy less than 0.9.

Mol	Type	Chain	Res	Atoms	RSCC	RSR	B-factors(Å^2)	Q<0.9
2	CL	B	402	1/1	0.92	0.17	51,51,51,51	0
2	CL	B	403	1/1	0.97	0.18	48,48,48,48	0
2	CL	B	401	1/1	0.97	0.27	45,45,45,45	0
2	CL	A	401	1/1	0.98	0.23	36,36,36,36	0
2	CL	A	402	1/1	0.99	0.23	40,40,40,40	0

6.5 Other polymers (i)

There are no such residues in this entry.

CONFIDENTIAL VALIDATION REPORT
