# Supplementary material for: Structures of three MORN repeat proteins and a re-evaluation of the proposed lipid-binding properties of MORN repeats
Source: PLoS One. 2020 Dec 9;15(12):e0242677. doi: 10.1371/journal.pone.0242677 (PMC7725318; doi:10.1371/journal.pone.0242677)
Supplement: S5 File — (PDF) [file pone.0242677.s023.pdf]

Full wwPDB X-ray Structure Validation Report (i)

Oct 18, 2019 – 08:28 pm BST

PDB ID	:	6T6Q
Title	:	Crystal structure of Toxoplasma gondii Morn1 (extended conformation).
Deposited on	:	2019-10-18
Resolution	:	2.90 Å(reported)

This is a Full wwPDB X-ray Structure Validation Report.

This report is produced by the wwPDB biocuration pipeline after annotation of the structure.

We welcome your comments at *validation@mail.wwpdb.org*
A user guide is available at
https://www.wwpdb.org/validation/2017/XrayValidationReportHelp
with specific help available everywhere you see the (i) symbol.

The following versions of software and data (see references (i)) were used in the production of this report:

MolProbity	:	4.02b-467
Xtriage (Phenix)	:	1.13
EDS	:	2.5
Percentile statistics	:	20171227.v01 (using entries in the PDB archive December 27th 2017)
Refmac	:	5.8.0158
CCP4	:	7.0 (Gargrove)
Ideal geometry (proteins)	:	Engh & Huber (2001)
Ideal geometry (DNA, RNA)	:	Parkinson et al. (1996)
Validation Pipeline (wwPDB-VP)	:	2.5

CONFIDENTIAL VALIDATION REPORT

1 Overall quality at a glance (i)

The following experimental techniques were used to determine the structure:
X-RAY DIFFRACTION

The reported resolution of this entry is 2.90 Å.

Percentile scores (ranging between 0-100) for global validation metrics of the entry are shown in the following graphic. The table shows the number of entries on which the scores are based.

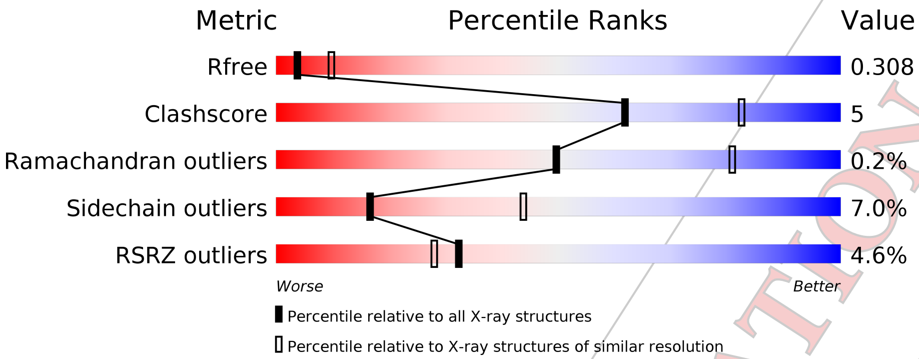

Metric	Whole archive (#Entries)	Similar resolution (#Entries, resolution range(Å))
R_{free}	111664	1716 (2.90-2.90)
Clashscore	122126	1924 (2.90-2.90)
Ramachandran outliers	120053	1884 (2.90-2.90)
Sidechain outliers	120020	1886 (2.90-2.90)
RSRZ outliers	108989	1669 (2.90-2.90)

The table below summarises the geometric issues observed across the polymeric chains and their fit to the electron density. The red, orange, yellow and green segments on the lower bar indicate the fraction of residues that contain outliers for >=3, 2, 1 and 0 types of geometric quality criteria. A grey segment represents the fraction of residues that are not modelled. The numeric value for each fraction is indicated below the corresponding segment, with a dot representing fractions <=5% The upper red bar (where present) indicates the fraction of residues that have poor fit to the electron density. The numeric value is given above the bar.

Mol	Chain	Length	Quality of chain
1	AAA	220	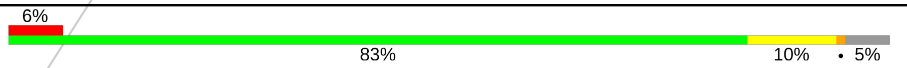
1	BBB	220	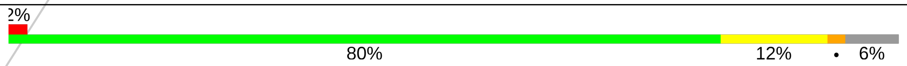

CONFIDENTIAL VALIDATION REPORT

2 Entry composition (i)

There is only 1 type of molecule in this entry. The entry contains 6333 atoms, of which 3014 are hydrogens and 0 are deuteriums.

In the tables below, the ZeroOcc column contains the number of atoms modelled with zero occupancy, the AltConf column contains the number of residues with at least one atom in alternate conformation and the Trace column contains the number of residues modelled with at most 2 atoms.

- Molecule 1 is a protein called Membrane occupation and recognition nexus protein MORN1.

Mol	Chain	Residues	Atoms						ZeroOcc	AltConf	Trace
			Total	C	H	N	O	S			
1	AAA	209	3181	1050	1513	289	323	6	76	0	0
1	BBB	207	3152	1041	1501	286	318	6	74	0	0

There are 8 discrepancies between the modelled and reference sequences:

Chain	Residue	Modelled	Actual	Comment	Reference
AAA	144	GLY	-	expression tag	UNP A0A0F7VBC6
AAA	145	PRO	-	expression tag	UNP A0A0F7VBC6
AAA	146	ALA	-	expression tag	UNP A0A0F7VBC6
AAA	147	MET	-	expression tag	UNP A0A0F7VBC6
BBB	144	GLY	-	expression tag	UNP A0A0F7VBC6
BBB	145	PRO	-	expression tag	UNP A0A0F7VBC6
BBB	146	ALA	-	expression tag	UNP A0A0F7VBC6
BBB	147	MET	-	expression tag	UNP A0A0F7VBC6

CONFIDENTIAL VALIDATION REPORT

3 Residue-property plots ⓘ

These plots are drawn for all protein, RNA and DNA chains in the entry. The first graphic for a chain summarises the proportions of the various outlier classes displayed in the second graphic. The second graphic shows the sequence view annotated by issues in geometryand electron density. Residues are color-coded according to the number of geometric quality criteria for which they contain at least one outlier: green = 0, yellow = 1, orange = 2 and red = 3 or more. A red dot above a residue indicates a poor fit to the electron density (RSRZ > 2). Stretches of 2 or more consecutive residues without any outlier are shown as a green connector. Residues present in the sample, but not in the model, are shown in grey.

- Molecule 1: Membrane occupation and recognition nexus protein MORN1

Chain AAA:

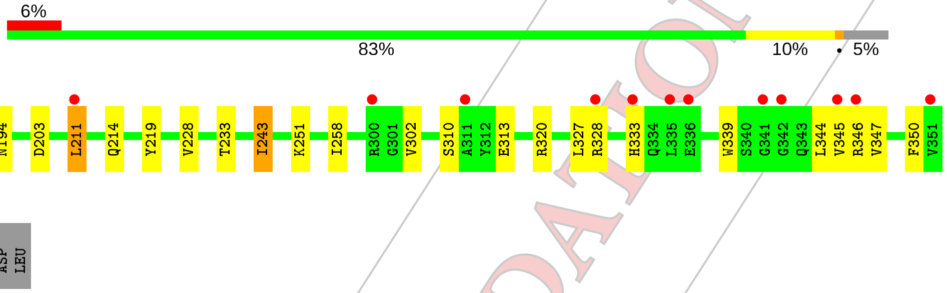

- Molecule 1: Membrane occupation and recognition nexus protein MORN1

Chain BBB:

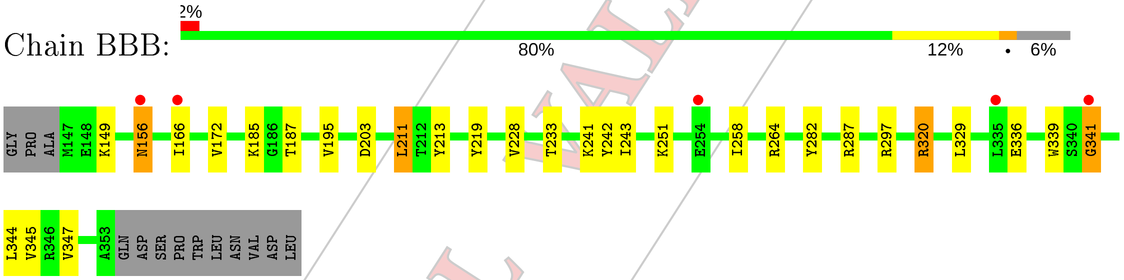

CONFIDENTIAL VALIDATION REPORT

4 Data and refinement statistics (i)

Property	Value	Source
Space group	P 43 21 2	Depositor
Cell constants<br>a, b, c, α, β, γ	53.92Å 53.92Å 348.85Å<br>90.00° 90.00° 90.00°	Depositor
Resolution (Å)	48.92 – 2.90<br>48.91 – 2.90	Depositor<br>EDS
% Data completeness<br>(in resolution range)	98.9 (48.92-2.90)<br>98.9 (48.91-2.90)	Depositor<br>EDS
R_{merge}	0.14	Depositor
R_{sym}	(Not available)	Depositor
$< I/\sigma(I) >$ [1]	1.68 (at 2.91Å)	Xtriage
Refinement program	REFMAC 5.8.0257	Depositor
R, R_{free}	0.276 , 0.311<br>0.276 , 0.308	Depositor<br>DCC
R_{free} test set	593 reflections (4.84%)	wwPDB-VP
Wilson B-factor (Å^2)	72.7	Xtriage
Anisotropy	0.934	Xtriage
Bulk solvent k_{sol}(e/Å^3), B_{sol}(Å^2)	0.34 , 42.3	EDS
L-test for twinning[2]	$< \|L\| > = 0.47$, $< L^2 > = 0.30$	Xtriage
Estimated twinning fraction	No twinning to report.	Xtriage
F_o,F_c correlation	0.94	EDS
Total number of atoms	6333	wwPDB-VP
Average B, all atoms (Å^2)	94.0	wwPDB-VP

Xtriage's analysis on translational NCS is as follows: *The largest off-origin peak in the Patterson function is 6.46% of the height of the origin peak. No significant pseudotranslation is detected.*

[1] Intensities estimated from amplitudes.

[2] Theoretical values of $< |L| >$, $< L^2 >$ for acentric reflections are 0.5, 0.333 respectively for untwinned datasets, and 0.375, 0.2 for perfectly twinned datasets.

CONFIDENTIAL VALIDATION REPORT

5 Model quality (i)

5.1 Standard geometry (i)

The Z score for a bond length (or angle) is the number of standard deviations the observed value is removed from the expected value. A bond length (or angle) with $|Z| > 5$ is considered an outlier worth inspection. RMSZ is the root-mean-square of all Z scores of the bond lengths (or angles).

Mol	Chain	Bond lengths		Bond angles	
		RMSZ	#$\|Z\|$ >5	RMSZ	#$\|Z\|$ >5
1	AAA	0.63	0/1716	0.74	0/2307
1	BBB	0.63	0/1699	0.74	0/2284
All	All	0.63	0/3415	0.74	0/4591

There are no bond length outliers.

There are no bond angle outliers.

There are no chirality outliers.

There are no planarity outliers.

5.2 Too-close contacts (i)

In the following table, the Non-H and H(model) columns list the number of non-hydrogen atoms and hydrogen atoms in the chain respectively. The H(added) column lists the number of hydrogen atoms added and optimized by MolProbity. The Clashes column lists the number of clashes within the asymmetric unit, whereas Symm-Clashes lists symmetry related clashes.

Mol	Chain	Non-H	H(model)	H(added)	Clashes	Symm-Clashes
1	AAA	1668	1513	1505	15	0
1	BBB	1651	1501	1493	19	0
All	All	3319	3014	2998	30	0

The all-atom clashscore is defined as the number of clashes found per 1000 atoms (including hydrogen atoms). The all-atom clashscore for this structure is 5.

All (30) close contacts within the same asymmetric unit are listed below, sorted by their clash magnitude.

Atom-1	Atom-2	Interatomic distance (Å)	Clash overlap (Å)
1:AAA:310:SER:HA	1:AAA:328:ARG:O	1.72	0.88
1:AAA:219:TYR:CZ	1:AAA:228:VAL:HG13	2.24	0.73

Continued on next page...

CONFIDENTIAL VALIDATION REPORT

Continued from previous page...

Atom-1	Atom-2	Interatomic distance (Å)	Clash overlap (Å)
1:BBB:219:TYR:CZ	1:BBB:228:VAL:HG13	2.28	0.69
1:AAA:302:VAL:HG22	1:AAA:313:GLU:HG2	1.77	0.66
1:AAA:344:LEU:HD12	1:BBB:347:VAL:CG2	2.31	0.61
1:AAA:347:VAL:HB	1:BBB:344:LEU:HD12	1.83	0.59
1:BBB:233:THR:HG23	1:BBB:243:ILE:HG13	1.86	0.58
1:BBB:211:LEU:HD12	1:BBB:213:TYR:CE1	2.40	0.56
1:AAA:344:LEU:HD12	1:BBB:347:VAL:HG21	1.88	0.55
1:AAA:339:TRP:CE2	1:AAA:344:LEU:HD23	2.41	0.55
1:BBB:339:TRP:CE2	1:BBB:344:LEU:HD23	2.44	0.53
1:AAA:211:LEU:C	1:AAA:211:LEU:HD12	2.29	0.53
1:BBB:344:LEU:HD13	1:BBB:345:VAL:N	2.25	0.52
1:AAA:344:LEU:HD13	1:AAA:345:VAL:N	2.25	0.52
1:BBB:149:LYS:HG3	1:BBB:166:ILE:HB	1.93	0.51
1:BBB:282:TYR:OH	1:BBB:297:ARG:NH1	2.45	0.49
1:BBB:219:TYR:CZ	1:BBB:228:VAL:CG1	2.96	0.48
1:AAA:156:ASN:HA	1:AAA:156:ASN:HD22	1.58	0.47
1:BBB:258:ILE:HG23	1:BBB:264:ARG:HG2	1.97	0.47
1:AAA:219:TYR:CZ	1:AAA:228:VAL:CG1	2.97	0.45
1:AAA:344:LEU:C	1:AAA:344:LEU:HD13	2.38	0.45
1:AAA:333:HIS:CE1	1:BBB:329:LEU:HD23	2.52	0.44
1:BBB:344:LEU:HD13	1:BBB:344:LEU:C	2.38	0.44
1:AAA:194:ASN:OD1	1:AAA:214:GLN:OE1	2.35	0.44
1:BBB:211:LEU:HD12	1:BBB:213:TYR:CD1	2.52	0.44
1:BBB:320:ARG:HB2	1:BBB:341:GLY:O	2.18	0.44
1:AAA:233:THR:HG23	1:AAA:243:ILE:HG22	1.99	0.43
1:BBB:156:ASN:HA	1:BBB:156:ASN:HD22	1.60	0.42
1:BBB:241:LYS:HE2	1:BBB:243:ILE:HD11	2.00	0.42
1:BBB:242:TYR:CD1	1:BBB:251:LYS:HD3	2.55	0.41

There are no symmetry-related clashes.

5.3 Torsion angles (i)

5.3.1 Protein backbone (i)

In the following table, the Percentiles column shows the percent Ramachandran outliers of the chain as a percentile score with respect to all X-ray entries followed by that with respect to entries of similar resolution.

The Analysed column shows the number of residues for which the backbone conformation was analysed, and the total number of residues.

CONFIDENTIAL VALIDATION REPORT

Mol	Chain	Analysed	Favoured	Allowed	Outliers	Percentiles	
1	AAA	207/220 (94%)	199 (96%)	8 (4%)	0	100	100
1	BBB	205/220 (93%)	202 (98%)	2 (1%)	1 (0%)	31	65
All	All	412/440 (94%)	401 (97%)	10 (2%)	1 (0%)	49	81

All (1) Ramachandran outliers are listed below:

Mol	Chain	Res	Type
1	BBB	341	GLY

5.3.2 Protein sidechains (i)

In the following table, the Percentiles column shows the percent sidechain outliers of the chain as a percentile score with respect to all X-ray entries followed by that with respect to entries of similar resolution.

The Analysed column shows the number of residues for which the sidechain conformation was analysed, and the total number of residues.

Mol	Chain	Analysed	Rotameric	Outliers	Percentiles	
1	AAA	159/168 (95%)	147 (92%)	12 (8%)	15	40
1	BBB	157/168 (94%)	147 (94%)	10 (6%)	19	49
All	All	316/336 (94%)	294 (93%)	22 (7%)	16	44

All (22) residues with a non-rotameric sidechain are listed below:

Mol	Chain	Res	Type
1	AAA	156	ASN
1	AAA	172	VAL
1	AAA	203	ASP
1	AAA	211	LEU
1	AAA	243	ILE
1	AAA	251	LYS
1	AAA	258	ILE
1	AAA	320	ARG
1	AAA	327	LEU
1	AAA	346	ARG
1	AAA	350	PHE
1	AAA	354	GLN
1	BBB	156	ASN
1	BBB	172	VAL
1	BBB	185	LYS

Continued on next page...

CONFIDENTIAL VALIDATION REPORT

Continued from previous page...

Mol	Chain	Res	Type
1	BBB	187	THR
1	BBB	195	VAL
1	BBB	203	ASP
1	BBB	211	LEU
1	BBB	287	ARG
1	BBB	320	ARG
1	BBB	336	GLU

Some sidechains can be flipped to improve hydrogen bonding and reduce clashes. There are no such sidechains identified.

5.3.3 RNA (i)

There are no RNA molecules in this entry.

5.4 Non-standard residues in protein, DNA, RNA chains (i)

There are no non-standard protein/DNA/RNA residues in this entry.

5.5 Carbohydrates (i)

There are no carbohydrates in this entry.

5.6 Ligand geometry (i)

There are no ligands in this entry.

5.7 Other polymers (i)

There are no such residues in this entry.

5.8 Polymer linkage issues (i)

There are no chain breaks in this entry.

CONFIDENTIAL VALIDATION REPORT

6 Fit of model and data (i)

6.1 Protein, DNA and RNA chains (i)

In the following table, the column labelled '#RSRZ> 2' contains the number (and percentage) of RSRZ outliers, followed by percent RSRZ outliers for the chain as percentile scores relative to all X-ray entries and entries of similar resolution. The OWAB column contains the minimum, median, 95th percentile and maximum values of the occupancy-weighted average B-factor per residue. The column labelled 'Q< 0.9' lists the number of (and percentage) of residues with an average occupancy less than 0.9.

Mol	Chain	Analysed	<RSRZ>	#RSRZ>2			OWAB(Å^2)	Q<0.9
1	AAA	209/220 (95%)	0.50	14 (6%)	18	13	72, 88, 118, 148	0
1	BBB	207/220 (94%)	0.50	5 (2%)	59	56	75, 96, 116, 128	0
All	All	416/440 (94%)	0.50	19 (4%)	32	28	72, 92, 118, 148	0

All (19) RSRZ outliers are listed below:

Mol	Chain	Res	Type	RSRZ
1	AAA	342	GLY	3.0
1	AAA	354	GLN	2.9
1	AAA	346	ARG	2.9
1	AAA	328	ARG	2.7
1	AAA	355	ASP	2.5
1	BBB	156	ASN	2.5
1	AAA	335	LEU	2.5
1	AAA	351	VAL	2.5
1	BBB	166	ILE	2.5
1	AAA	341	GLY	2.5
1	BBB	335	LEU	2.4
1	AAA	311	ALA	2.3
1	AAA	211	LEU	2.3
1	AAA	300	ARG	2.2
1	AAA	333	HIS	2.2
1	AAA	345	VAL	2.2
1	AAA	336	GLU	2.2
1	BBB	341	GLY	2.1
1	BBB	254	GLU	2.1

6.2 Non-standard residues in protein, DNA, RNA chains (i)

There are no non-standard protein/DNA/RNA residues in this entry.

CONFIDENTIAL VALIDATION REPORT

6.3 Carbohydrates (i)

There are no carbohydrates in this entry.

6.4 Ligands (i)

There are no ligands in this entry.

6.5 Other polymers (i)

There are no such residues in this entry.

CONFIDENTIAL VALIDATION REPORT
