# Supplementary material for: Structures of three MORN repeat proteins and a re-evaluation of the proposed lipid-binding properties of MORN repeats
Source: PLoS One. 2020 Dec 9;15(12):e0242677. doi: 10.1371/journal.pone.0242677 (PMC7725318; doi:10.1371/journal.pone.0242677)
Supplement: Attachment — Submitted filename: 103458_1_rebuttal_1778131_qbrxgw.pdf [file pone.0242677.s024.pdf]

RE: Review Commons Refereed Preprint #RC-2020-00173
"Structures of three MORN repeat proteins and a re-evaluation of the proposed lipid-binding properties of MORN repeats"

Response to Reviewers
The response to reviewers consists of three parts:

1. A summary of the main points from the two reviews, and the authors' response to these points.
2. A detailed revision plan for the preprint, taking into account both the main points of the reviews, and other comments made by the reviewers.
3. A point-by-point response to the reviewers.

For figure citations, OV = old version, i.e. bioRxiv preprint 2019-826180v2, and NV = new version, i.e. revised and re-submitted version.

<u>1. Summary of main points by the reviewers, and authors' responses:</u>

- Both reviewers felt that the manuscript was overlong; Reviewer 1 recommended either shortening it or splitting it into two stories, while Reviewer 2 recommended cutting down the text.
    - We have considerably shortened the manuscript in accordance with this request (see revision plan below). We had already considered splitting the manuscript into two parts during the drafting stage, and had rejected this possibility as the data are intertwined - the retroactive validation of the dimer interface by the mutagenesis constructs (OV Fig. S3 [NV Fig. S4]) being a good example.
    - The revised manuscript features 7 main figures and 13 supplementals.
- Both reviewers felt too much text and figure space was allocated to negative data, specifically the investigation of potential lipid binding by the TbMORN1 protein, and that there should be more focus on the positive parts of the story.
    - A key part of shortening the manuscript has been moving most of the negative data on lipid binding into the supplemental figures, and considerably shortening the associated text. This has allowed the main figures and associated text to focus more on the positive elements of the project, while still ensuring publication of all the data.
- The reviewers appear to be in slight disagreement concerning discussion of the data. Reviewer 1 has encouraged more speculation on the physiological role of PE binding, a potential lipid transfer function, a role for calcium ions, the relevance of the observed disulphide bond, and the role of zinc ions in apicomplexan proteins; Reviewer 2 has recommended avoiding excessive speculation or inference.
    - Given that both reviewers have agreed that the original manuscript was overlong, we have implemented Reviewer 2's suggestion here and reduced the amount of speculation in the revised text.
- The reviewers agreed that the technical quality of the data was high and that the conclusions drawn were robust.

- We are glad that the reviewers were appreciative of the data quality. For this reason, we were reluctant to remove any of the data from the manuscript and would prefer instead to transfer it to the supplementals. We feel that the negative data still have considerable community value, given that they show that MORN repeats are not automatically lipid-binding modules and can thus act as a caveat to other researchers.

2. Detailed revision plan for the preprint:

- We have implemented the reviewers' suggestions and substantially shortened the manuscript, primarily by trimming the (phospho)lipid-binding section, which contains a large amount of negative data. The following main figures have been moved into the supplemental section:
    - OV Fig. 2 ("TbMORN1 interacts with phospholipids but not liposomes") has become NV Fig. S2
    - OV Fig. 4 ("TbMORN1(2-15) does not bind to liposomes in vitro") has become NV Fig. S6
    - OV Fig. 8 ("Conservation and properties of residues in TbMORN1(7-15)") has become NV Fig. S11
  - This has left a total of 7 main figures and 13 supplementals.
  - The text associated with the entirety of the lipid-binding part (OV lines 210-530, OV Figs. 2-6 [NV Figs. 2-4, S2, S6], OV Supplemental Figs. 2-6 [NV Supplemental Figs. S3-S5, S7, S8]) has been condensed. The focus of this section is now on the positive parts of the data: the PE association (OV Fig. 3 [NV Fig. 2]) and the in vivo work (OV Figs. 5, 6 [NV Figs. 3, 4]).
  - We have additionally limited the amount of inference and speculation in the manuscript.

3. Point-by-point responses to the reviewers

The authors' responses are shown in *red italics.*

Reviewer #1 (Evidence, reproducibility and clarity (Required)):

MORN (membrane occupation and recognition nexus) repeat proteins are found in prokaryotes and eukaryotes. They feature characteristic repeats in their primary sequence, have been assumed to play a role in lipid binding, but remain poorly characterized on the functional and structural level. This manuscript tries to address both these questions and is organized in major parts. In the first part the authors characterize a putative role of MORN repeat proteins in lipid binding and membrane association. In the second part, the authors use X-ray crystallography to establish the structure of MORN repeat proteins and to investigate the dimerization.

As a cleverly chosen point of departure, they focus their study particularly on MORN1 from Trypanosoma brucei (TbMORN1), which is composed solely on MORN repeats. The structures of MORN repeats (from several species) in part two provide interesting insights

into their mode of homotypic interactions and their role as dimerization or oligomerization devices.

The lipid binding and membrane association of MORN proteins in the first part remains somewhat confusing and unclear, despite the use of a whole battery of techniques.
We anticipate that the shortening and refocusing of the lipid binding data has addressed this issue.

It is questionably, why the authors invest so many figures and words to inform the reader on negative results.
We have chosen to publicise our negative data in full because, as noted in the manuscript, there is a widespread and erroneous assumption that MORN repeats are lipid binding modules. We feel that publishing these data will allow them to act as a caveat to other researchers working on MORN repeat proteins. We have, however, addressed the reviewer's request in that we have considerably shortened the text associated with these data and have moved the corresponding figures into the supplementals.

The authors suggest that MORN proteins can bind to lipids via their hydrophobic acyl chains-which is 'very hard to imagine under physiological conditions unless TbMORN1 is a lipid carrier and not a membrane-binding proteins. Unfortunately, a role as lipid carrier has not been rigorously tested.
The reviewer is correct that we have not specifically tested for a function as a lipid carrier protein and although this was only speculation, it has been toned down accordingly.

In this sense the first part remains somewhat immature and incoherent. Furthermore, they suggest based on the lack-of-evidence that MORN proteins do not bind membranes in vivo and in vitro.
We are not clear where this suggestion was made. Our data indicate that TbMORN1 does not directly bind membranes in vivo or in vitro, and we therefore noted that putative lipid binding by other MORN repeat proteins should be viewed with caution. Specifically, we stated in the Discussion (OV lines 955-956) that "the presence of MORN repeats in a protein should not be taken as indicative of lipid binding or lipid membrane binding without experimental evidence". Again, our expectation is that the major changes planned for the data presentation in this section will make it more coherent.

The main issue of this manuscript is, in my view, the way the data were presented.The manuscript is generally well-written, but much too long. The structural work is important and concise.
We have considerably shortened the manuscript as per the reviewer's request, and especially the section on lipid binding.

The first part, however, reports in five separate figures on a lack of membrane binding by a MORN protein and its ability to bind individual lipids. The physiologically relevance of this lipid binding is questionable as acknowledged by the authors.
We have moved two of these figures (OV Figs. 2, 4) into the supplementals section [NV Figs. S2, S6], shortened the associated text, and limited the amount of speculation.

Even though I find it important that the membrane/lipid binding ability of MORN proteins is rigorously tested, I would highly recommend to separate the current manuscript in two independent stories. Alternatively, I would recommend to reduce the first part into a single figure and to remove the most artifactual assays.
We have implemented the second of these two suggestions for the manuscript. We had already considered splitting the manuscript during the drafting stage, but rejected this possibility as the data were too intertwined. Consequently, we have opted to considerably reduce the first part, and moved OV Figs. 2 and 4 into the supplementals [NV Figs. S2, S6]. We would prefer not to remove data altogether as they are likely to have community value even if they are negative and as noted, they are of good quality.

In the current form, the first part and the second part of the manuscript remain somewhat detached from each other. The characterization of the lipid binding/membrane binding properties has a number of substantial weaknesses (e.g. use of quite different, non-physiological buffers for membrane binding assays; use of deletion mutants for the binding assays, which do not show the full potential of oligomerization). This which makes it hard to read and confuses the reader. Even though I have no reason to doubt the conclusions by the authors, I do not think that all necessary caution has been invested to rule out other possibilities.
We believe that the shortening and refocusing of the manuscript should address these issues. For consideration of the buffer and deletion mutant points, please see responses to Major Points below.

In summary, even though the technical quality of the individual performed assays is high, there are some conceptual issues that make it hard to make a strong case based on a collection of individual, clear datasets. Even though I find the structures of the MORN proteins important, timely, and interesting, I would not recommend this study for publication in its current form. The manuscript would be more fun to read if both of the parts would be shortened substantially and more focused.
We have implemented this suggestion: the manuscript has been considerably shortened (from 20,489/135,073 to 18,555/103,988 characters/words, focused on reducing the negative lipid-binding results).

While I agree that most evidence provided on lipid/membrane binding of TbMORN1 argue against a direct role of MORN proteins in membrane binding, I feel that the experimental approach is not coherent enough. See a few major points of criticism below.

****Major Points:****

1) The authors decide to characterize the membrane binding of a MORN repeat protein using a deletion variant that lacks the N-terminal repeat. However, in Figure 1B they show that the N-terminal repeat is important for the formation of higher-order oligomers. While I fully understand that the presence of the most N-terminal repeat does hamper the structural work, I find it problematic to remove it for the lipid/membrane-binding assays. The formation of

higher oligomeric species beyond the dimer, may be important for membrane binding/recruitment (avidity effects).

As we explained in the manuscript, the reason for not using the full-length protein for in vitro work was because it was polydisperse, and that the yields were extremely low. See OV lines 178-179 ("The yields of TbMORN1(1-15) were always very low, making this construct not generally suitable for in vitro assays".) and OV lines 411-414 ("...TbMORN1(1-15), which was polydisperse in vitro and formed large oligomers (Fig. 1B). The membrane-binding activity of these polydisperse oligomers was not possible to test in vitro, as the purification yields of TbMORN1(1-15) were always low."). Consequently, we used the longest construct that was suitable in terms of chemical and oligomeric homogeneity. Using the full-length protein would have had inherent problems with aggregation, and consequently would have compromised the data and derived results. In order to make this clear in the manuscript we edited the sentence mentioned above as follows:

"It was not possible to test the membrane-binding activity of these polydisperse oligomers in vitro however, as the purification yields of TbMORN1(1-15) were always low. As an alternative, the possible membrane association of TbMORN1(1-15) was examined in vivo."

2) (Related to point 1) I do not understand the choice of the buffers used for some of the assays. The use of pH 8.5 and NaCl concentrations of 200 mM are non-physiological.

These were the buffer conditions required to retain the protein in a monodisperse state, suitable for in vitro assays.

For CD spectroscopy, a high ionic strength was obtained by the use of 200 mM NaF. If a high ionic strength is required to prevent the formation of higher oligomers of MORN, it raises the question if the formation of higher oligomers (under physiological conditions) may also contribute to their function.

The oligomers of TbMORN1 may indeed be the most functionally relevant form of TbMORN1 but we do not currently have a means of testing this in vitro, as acknowledged in the text (OV lines 411-414, quoted above). The aim of CD spectroscopy was to assess fold integrity and stability of different constructs; we used buffers as recommended for the CD spectroscopy experiments by Kelly et al, 2005 (doi:10.1016/j.bbapap.2005.06.005) (Table 1 and section 4.2). Furthermore, the CD spectra of TbMORN(1-15) and TbMORN(2-15) (OV Fig. S1E [NV Fig. S1E]) are basically superimposable, suggesting identical secondary structure content at the concentration used for these experiments.

It is unclear, in which buffer the fluorescence anisotropy measurements were performed.

We have provided details on the buffer conditions for the fluorescence anisotropy experiments in the Materials and Methods section, NV page 23, lines 962-963.

The sucrose-loaded vesicles were hydrated in a 20 mM HEPES pH 7.4, 0.3 M Sucrose. The composition of the buffer after the addition of MORN proteins is not clear.

The Materials and Methods are now unambiguous on this point. Please see NV lines 1036-1046: "6 µM Rhodamine B dihexadecanoyl phosphoethanolamine (Rh-DHPE) was added to all lipid mixtures to facilitate the visualisation of the SLVs. The lipid mixtures were dried under a nitrogen stream, and the lipid films hydrated in 20 mM HEPES pH 7.4; 0.3 M sucrose. The

lipid mixtures were subjected to 4 cycles of freezing in liquid nitrogen followed by thawing in a sonicating water bath at RT. The vesicles were pelleted by centrifugation (250,000 × g, 30 min, RT) and resuspended in 20 mM HEPES pH 7.4, 100 mM KCl to a total lipid concentration of 1 mM. SLVs were incubated with 1.5 µM purified TbMORN1(2-15) in gel filtration buffer (20 mM Tris-HCl pH 8.5, 200 mM NaCl, 2% glycerol, 1 mM DTT) at a 1:1 ratio (30 min, RT)." The liposomes were at physiological pH and close to physiological ionic strength.

Despite the use of an impressive array of techniques, this first part of the manuscript remains somewhat immature and incoherent. Due to the use of constructs that have not the full ability to oligomerize (point 1) and due to the inconsistent use of experimental conditions, it is hard to draw firm conclusions from this first part.
Any biochemical study is conducted within the constraints of the choice of construct and the choice of buffer conditions, and the data are valid within those parameters. This applies as much to positive data as to negative data, so we are not clear why the reviewer is placing such emphasis on this point. In the case of the LiMA data, which are the most unbiased and comprehensive dataset in the manuscript, these experiments were well-controlled and there were also domains present that were recruited to membranes under the buffer conditions, allowing us to rule out that the assay conditions were completely unsuitable. Validating negative results should be done as carefully and with as many orthogonal approaches as the validation of positive results. The reviewer acknowledges below that "the data point in the direction that MORN proteins (or at least TbMORN1) does not directly bind to membranes". This is the conclusion that we wanted to communicate.

For example: In Figure 2E TbMORN(2-15) does show some concentration-dependent binding, which -however- is interpreted as background binding. What are the results using this assay (or better: a liposome floatation assay) when using full-length TbMORN(1-15) in a more physiological buffer?
As noted already, it is not possible to use the TbMORN1(1-15) construct for in vitro assays owing to the extremely low yields and polydisperse nature of the protein. The excess full-length protein was associated with the cytosolic fraction and not the membrane fraction in vivo (OV Fig. 6B [NV Fig. 4B]).

The statement that MORN proteins bind to lipids, but not to liposomes/membranes is -in my view- not sufficiently addressed to make a strong case.
At no point do we suggest that MORN repeat proteins in general bind to lipids and not to liposomes/membranes. On the contrary, and as detailed in the manuscript, we set out to assay the lipid binding activity of TbMORN1, found that it appears to bind to lipids but not to liposomes/membranes, and have therefore cautioned that lipid or liposome/membrane binding of other MORN repeat proteins must be tested experimentally before claims of function are made.

3) The physiological relevance of lipid binding to MORN proteins remains obscure (as also acknowledged by the authors). Does the binding of PE lipids to the MORN protein have a physiological role? Does the binding of fluorescent PI(4,5)P2 point to a physiological role of MORN proteins?

These are interesting questions that we would like to address in future work.

4) In light of recent data from the Chris Stefan lab (PMID: 31402097) a co-incidence detection of PI(4,5)P2, PS, and cholesterol seems possible. Can the authors address this possibility?
Again, the involvement of cholesterol, PS, and PI(4,5)P_2 would be interesting questions for subsequent work but are beyond the scope of the present study. We did partially address this issue in our use of PI(4,5)P_2, POPC and cholesterol containing liposomes in liposome cosedimentation assays, which showed no binding (OV Fig. S3A [NV Fig. S4A]).

Furthermore, the role of Ca2+ signaling / Ca2+ ions has not been addressed. In light of the important role of Ca2+ for the recognition of PI(4,5)P2 (PMID: 28177616), this point should be addressed.
We carried out liposome pelleting assays in the presence of Ca^{2+} and Mg^{2+}, and saw no binding by TbMORN1(2-15) in either condition (see data below). These data were not included in the MS because of the insufficient number of technical replicates available.

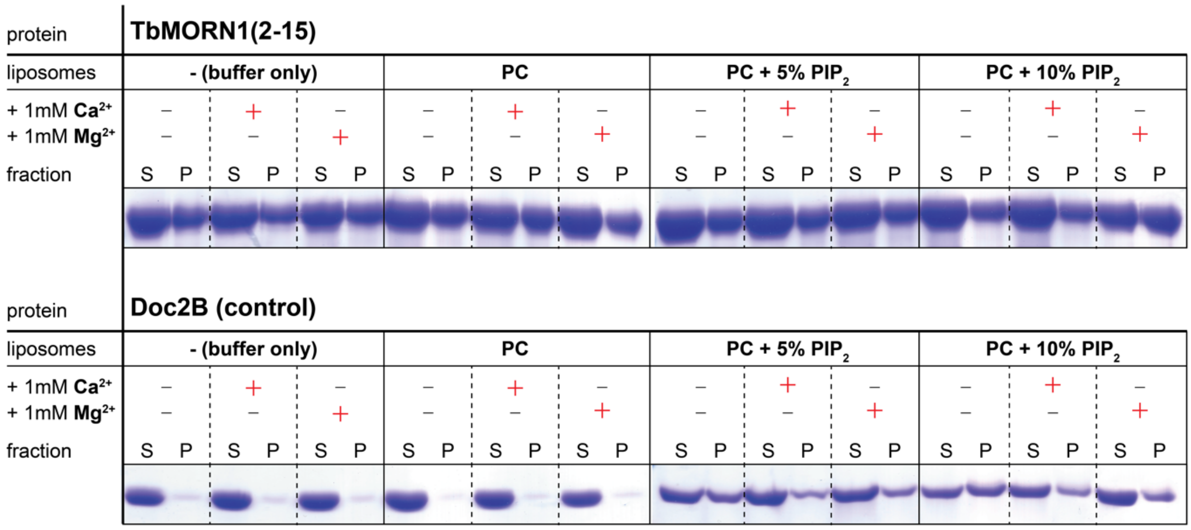

5) For characterizing the binding of lipids to MORN proteins, the authors use non-physiological fluorescent and short-chain lipid analogues at concentrations, which are unlikely to occur for endogenous PIPs in the cytosol of cells. Why choosing such an artificial system? Why introducing this system at length, if other -less artifact-prone- assays are available? I would recommend to not feature this assay as prominently as it was in the current study.
Our aim was to stick to using the same fluorophore throughout all the experiments. The choice of short-chain lipids was constrained by what was commercially available with the BODIPY TMR fluorophore. We have implemented the reviewer's suggestion in the manuscript, and the text associated with the fluorescence anisotropy assays has been considerably shortened. We are aware that the chosen concentration of the fluorescent lipids was out of physiological range, but the requirements of the fluorescence anisotropy itself necessitated a compromise. The possible shortcomings of the fluorescence anisotropy assays are, we believe, more than amply compensated by the LiMA data.

6) How would PE find its way to the lipid binding region in MORN? Would it diffuse to the MORN protein via the aqueous phase or would the MORN protein pickup PE form membranes up collision? The authors should address this point, by separating the lipid-depleted MORN protein from donor-vesicles containing PE by a dialysis membrane. If PE would not find its way to the lipid binding site of MORN, this would imply that MORN protein can extract lipids only upon colliding with the membrane. What is the stoichiometry of PE to MORN?
These are all interesting questions that we would like to pursue in subsequent work, but we feel that they are beyond the scope of the present study. Until we have conditions suitable for obtaining high yields and monodisperse populations of the full-length protein, which probably also necessitates developing conditions for controlled oligomerisation, it would be premature to start this. As to how it picks up PE: it is well known that specific lipid binding/chaperoning proteins can deliver their lipid cargo to other proteins. Additionally, proteins that bind lipids use hydrophobic domains to both interact with and sequester fatty acids and/or lipids from membranes The literature is populated with lots of such examples. https://www.sciencedirect.com/science/article/pii/S0092867416310765.

Despite my critique raised above, I agree with the authors that the data point in the direction that MORN proteins (or at least TbMORN1) does not directly bind to membranes. Their data, however, would still be consistent with a role as lipid transfer protein and a recruitment of MORN proteins to the membrane by other proteins. Have the authors performed any additional experiments in this direction? Also, the potential role of palmitoylation is only mentioned in the discussion (page 22), while palmitoylation would provide a simple means for membrane recruitment.
We are glad that the reviewer concurs with our main conclusion. We agree, as noted in the discussion, that a role as a lipid transfer protein might still be possible, and this is something that we would like to pursue in follow-up work. We have not yet performed any additional experiments in this direction. Concerning palmitoylation, the predictions using the CSS-Palm software were always weak and ambiguous, and in addition the best candidate cysteine residue was Cys351, which is in our structure engaged in the disulphide bond observed in the C2 crystal form. We feel that this is something to keep in mind, but is not yet a strong enough hypothesis to pursue intensively.

****Minor Points:****

Figure 1B: The authors should provide information on the void volume of the column.
Implemented in the figure legend (7.2 ml).

Page 17, line 696-701: The authors point out that the C2 crystal form is stabilized by two disulfide bridges. The authors should comment on the physiological relevance of these disulfide bridges.
Given the reducing environment of the cytosol, it is an open question as to whether these disulphide bridges exist in vivo. We would prefer not to speculate on this point, as we do not feel it would be productive.

Page 18, line 734-740: The authors should provide data on the potential role of Zn2+ on MORN function in a physiological context. The section describing that the dimer is stabilized by Zn2+ ions (pages 18 and 19) lacks a discussion if Zn2+ are functionally relevant. There is only a beautiful sequence analysis and a discussion of the conservation of the Zn2+ coordinating residues. Can the authors perform Zn2+ titrations and SEC-MALS experiments (or alternatives such as SAXS) to show that Zn2+ indeed affects the oligomeric state of only the PfMORN, but not the other MORN proteins that form alternative dimers?
The known requirement for zinc ions in Plasmodium growth was already noted (OV lines 992-993, Marvin et al., 2012), and is, we believe, sufficient to address the issue of physiological relevance at this stage. The zinc ions are predicted to affect the architecture of the apicomplexan (Plasmodium, Toxoplasma) MORN1 protein dimers, not their oligomeric state. For PfMORN1, SEC-MALS and SAXS were carried out in 20 mM Tris-HCl pH 7.5, 100 mM NaCl with no zinc present. When EDTA was added, no change in behaviour of the protein was seen by SEC-MALS. When "TPEN", a strong zinc chelator, was added, the protein precipitated in SEC-MALS experiments.

Reviewer #1 (Significance (Required)):

A putative role of MORN proteins in membrane and lipid binding is addressed. The view the MORN proteins bind directly to membranes is challenged. Structures of dimeric MORN proteins provide
important insight into the modes of dimerization.

There is a recent structure of MORN proteins (which is referenced by the authors), but I feel that additional structural work is important and justified. The work on membrane vs. lipid binding is important, but not sufficiently addressed in the current manuscript.
We are glad that the reviewer finds the structural work important and justified, although we disagree with the reviewer's assessment of the lipid binding. As noted in the previous paragraph, our data challenge the assumption that MORN repeat proteins directly bind membranes, and we feel that this alone is a significant conceptual advance.

I would recommend to separate the study in two parts. The audience is likely to confused (or bored) by the lengthy discussion on whether or not MORN proteins bind lipids and or membrane or not.
We would prefer to implement the reviewer's other suggestion, namely that the manuscript is considerably shortened and less focus given to the negative data on lipid binding.

I am not an expert in structural biology, but have a fair understanding of structural biology. I have worked on lipid binding proteins and have a very good understanding of lipid/membrane-binding assays.

Reviewer #2 (Evidence, reproducibility and clarity (Required)):

****Summary****

The manuscript describes an extensive and detailed investigation into the structure and function(s) of MORN domains. It has to be acknowledged that, despite the considerable amount of work reported, the conclusions are rather limited. From a technical viewpoint, the experiments have been appropriately executed and, generally, I concur with the conclusions drawn. However, the manuscript is over-long: in general, I would recommend concentrating on positive conclusions which can be drawn from the data and avoid excessive speculation or inference (some examples given below).
We are glad that the reviewer is satisfied with the technical quality of the work and (in general) the validity of the conclusions. We acknowledge that the original submission was fairly long, and have considerably shortened the revised manuscript and focused more on the positive conclusions in order to implement this suggestion.

****Major Comments****

There are three general- perhaps rather obvious- points to make. First, there is no particular reason to think that conservation of structure necessarily indicates conservation of a particular function. There seems to be an implicit assumption that MORN domains are associated with a specific, well-defined biological function. Given their diversity, are there particular reasons to think that this is the case?
The reviewer is exactly right that there is an implicit assumption that MORN domains are associated with a specific, well-defined function: specifically, lipid binding. It is this assumption, which has been widely circulated in the almost complete absence of experimental evidence, that we are challenging. We agree that MORN repeats are likely to be capable of multiple functions, and protein-protein interactions are now better supported than protein-lipid interactions.

Second, a strategy which examines the properties of just the recombinant MORN domains in vitro, removed from the context of the whole protein (eg junctophilin) or- importantly- its interacting partners in vivo, has obvious limitations. Frequently a reductionist approach is successful; however, in this case, MORN domains appear to be less tractable to that kind of approach. For all the in vitro binding and structural experiments presented, there is always a concern that the absence of other parts of the relevant MORN-containing protein or its partners could explain failure or inconsistency of in vitro biological activity measurements.
Again, the reviewer is right that there is an inherent contextual limitation to any in vitro work that utilises a single protein, but this is a concern that - by definition - could be raised about any in vitro study utilising a single protein. It should be noted that we have also carried out in vivo experiments using TbMORN1 (OV Figs. 5, 6 [NV Figs. 3, 4]).

Third, the possibility that MORN domains might mediate interactions with other proteins seems to be given little consideration, in spite of the Li et al (2019) paper. An experimental

strategy which looked for binding partners (eg by pulldown assay) might have provided more insight.

These data are already in the literature. A previous study by the same team (Morriswood et al., 2013) used proximity-dependent biotin identification to identify candidate binding partners and near neighbours of TbMORN1.

In order to stress this point we added the following sentence in the discussion section, NV pages 18-19, lines 774-778.

"The concluding data presented here suggest that TbMORN1 utilises this oligomerisation capacity to build mesh-like assemblies, which can reach considerable size in vitro (Fig. 7G). These mesh-like assemblies may reflect the endogenous organisation of the protein in vivo, where a number of binding partners have already been identified (Morriswood et al., 2013)".

****Minor Comments****

1. In the abstract and elsewhere the authors refer to a possible function of MORN domains as 'dimerisation and oligomerisation devices' (line 53). What is the evidence that dimer formation is important for function in vivo?

This is an interesting and important question and one that we would like to address in future work. We did attempt to generate trypanosome cell lines that inducibly expressed monomeric TbMORN1 (the double mutant, where the point mutations were simultaneously introduced in the dimerisation interface in repeats 13 and 14), but no expression of the ectopic protein was ever observed (9 separate clones obtained in 3 independent transfections). This might indicate the importance of the dimeric state in vivo, perhaps hinting that dimerisation is important for protection from degradation. In general, proteins assuming higher oligomeric states in homo- or heteromeric assemblies benefit from increased robustness in the cellular environment and optimised activity by the following means:

- *Increased stability by decreasing the surface area/volume ratio*
- *Simple construction of larger complexes*
- *Allosteric regulation*
- *Co-localisation of distinct biological functions*
- *Substrate channelling*
- *Protection from aggregation or degradation*

Which or which combination of the factors is relevant for TbMORN1 being a functional dimer in vivo is difficult to say at this point.

2. Did the authors attempt to co-crystallize TbMORN1(7-15) with PI(4,5)P2?

No. For crystallisation, we used lysine methylated samples, and by doing this we neutralised positively-charged potential binding sites which would have interacted with the negatively charged lipid headgroup. We did not observe any bound lipids in the electron density maps obtained from the crystals.

3. Fig 2C: did the authors also estimate binding stoichiometry as well as the equilibrium binding constants for these data? This should be determined by fitting a single binding site

model to the data. Other methods (eg ITC) can probably determine this with more accuracy. The value of stoichiometry is sometimes forgotten in such binding measurements- is one ligand bound per monomer or dimer, for example?

We discussed estimation of the binding stoichiometry in the fluorescence anisotropy assays at some length, but the conclusion was that the required experiments would contain too many approximations to provide high-confidence data. We did use ITC and also MST, but did not observe any binding with these assays.

4. Lines 674-678 I found it hard to work out whether these constructs harbour the natural C-terminal sequence without truncation or addition of an affinity tag. I think the answer is 'yes' but it was difficult working this out from the details in M&M.

*TbMORN1(7-15) crystallisation was with a C-terminal Strep tag; TgMORN1(7-15) and PfMORN1(7-15) had their affinity tags removed by protease treatment prior to crystallisation. We have clarified this point in the M&M, page 29, lines 1189-1192: "**Crystallisation of TbMORN1(7-15) (with a C-terminal Strep tag), TgMORN1(7-15) and PfMORN1(7-15) (both with affinity tags removed) was performed at 22 °C using a sitting-drop vapour diffusion technique and micro-dispensing liquid handling robots (Phoenix RE (Art Robbins Instruments) and Mosquito (TTP labtech)**."*

5. Lines 688-694 The PISA interface analysis is useful here in distinguishing crystal contacts from those which persist in solution. The discussion of the results is unclear, however, on this critical point: were the dimer interfaces the only contacts which were significant in the various crystal forms?

Yes, correct. PISA showed that the described dimerisation contacts were the only significant ones in the various crystal forms. Other crystals contacts had typically low P-values and poor ΔG and small "radar" surface in the complexive PISA analysis.

In the case of both TbMORN1 crystal forms and in the case of the TgMORN1 P43212 crystal form we have a dimer in the asymmetric unit, while in the case of the PfMORN1 and TgMORN1 P6222 form we have one molecule in the asymmetric unit, and the dimer is created by the crystallographic twofold axis. In the latter cases the quaternary structure resulting from the symmetry operations was the top-scoring one considering either P-values and/or the number of stabilising interactions buried surface area.

6. Lines 754-763 This paragraph seems rather speculative and is a good example where the text could be cut down.

If the line citation is correct, then we disagree with this assessment and would prefer not to implement it. The paragraph in question concerns a detailed and very precise discussion of the side chain interactions that stabilise the V-shaped forms of TgMORN1 and PfMORN1.

7. Line 765-788 This section is also rather overdone: such observations are only useful if they are subsequently tested by recording dimer conformation for a representative selection of MORN dimers from different species.

Again, we disagree with the reviewer's assessment of this analysis. The analysis has considerable predictive power and already has some experimental validation via the SAXS observation that PfMORN1 is capable of forming extended dimers in solution (OV Fig. 10C [NV Fig. 7C]).

8. Lines 800-801 I don't think this statement is strictly correct. The SAXS data show that PfMORN1(7-15) adopts an extended conformation, with no evidence of the 'V' shaped structure. Related to that point, from what I could glean from the SAXS Methods section, all solution conditions for these experiments were conducted without Zn2+? If some dimer interfaces require Zn2+, should it not be included?
We have clarified this statement. The SAXS experiments were conducted without zinc, and, as we have stressed, the V-shaped form of TgMORN1 and PfMORN1 was only ever observed in the crystals. For PfMORN1, SEC-MALS and SAXS were carried out in 20 mM Tris-HCl pH 7.5, 100 mM NaCl with no zinc present. When EDTA was added, no change in behaviour of the protein was seen by SEC-MALS. When "TPEN", a strong zinc chelator, was added, the protein precipitated in SEC-MALS experiments.

Reviewer #2 (Significance (Required)):

There is certainly value in establishing that MORN domains do not, in vitro, appear to bind to lipid vesicles, and to define their lipid binding capability (although it is rather complex). The crystal structures and SAXS data extend the rather limited structural data on MORN domains. Despite the effort involved, conclusions about likely functions of MORN domains in vivo are rather limited.
We are glad that the reviewer acknowledges the value in challenging the assumption that MORN repeats are lipid binding devices, and that the structural data are important for expanding the knowledge base on this class of repeat motif proteins. In vivo functional work is being actively pursued at present.

My expertise lies in X-ray crystallography and protein biochemistry.
